# OpenEP: A Cross-Platform Electroanatomic Mapping Data Format and Analysis Platform for Electrophysiology Research

**DOI:** 10.3389/fphys.2021.646023

**Published:** 2021-02-26

**Authors:** Steven E. Williams, Caroline H. Roney, Adam Connolly, Iain Sim, John Whitaker, Daniel O’Hare, Irum Kotadia, Louisa O’Neill, Cesare Corrado, Martin Bishop, Steven A. Niederer, Matt Wright, Mark O’Neill, Nick W. F. Linton

**Affiliations:** ^1^King’s College London, London, United Kingdom; ^2^Centre for Cardiovascular Science, The University of Edinburgh, Edinburgh, United Kingdom; ^3^Invicro, Ltd., London, United Kingdom; ^4^Guy’s and St Thomas’ NHS Foundation Trust, London, United Kingdom; ^5^Imperial College London, London, United Kingdom

**Keywords:** electroanatomic mapping, atrial fibrillation, data storage and retrieval, conduction velocity, ablation electrophysiology, contact force, electrophysiology – arrhythmia mapping and ablation

## Abstract

**Background:**

Electroanatomic mapping systems are used to support electrophysiology research. Data exported from these systems is stored in proprietary formats which are challenging to access and storage-space inefficient. No previous work has made available an open-source platform for parsing and interrogating this data in a standardized format. We therefore sought to develop a standardized, open-source data structure and associated computer code to store electroanatomic mapping data in a space-efficient and easily accessible manner.

**Methods:**

A data structure was defined capturing the available anatomic and electrical data. OpenEP, implemented in MATLAB, was developed to parse and interrogate this data. Functions are provided for analysis of chamber geometry, activation mapping, conduction velocity mapping, voltage mapping, ablation sites, and electrograms as well as visualization and input/output functions. Performance benchmarking for data import and storage was performed. Data import and analysis validation was performed for chamber geometry, activation mapping, voltage mapping and ablation representation. Finally, systematic analysis of electrophysiology literature was performed to determine the suitability of OpenEP for contemporary electrophysiology research.

**Results:**

The average time to parse clinical datasets was 400 ± 162 s per patient. OpenEP data was two orders of magnitude smaller than compressed clinical data (OpenEP: 20.5 ± 8.7 Mb, vs clinical: 1.46 ± 0.77 Gb). OpenEP-derived geometry metrics were correlated with the same clinical metrics (Area: *R*^2^ = 0.7726, *P* < 0.0001; Volume: *R*^2^ = 0.5179, *P* < 0.0001). Investigating the cause of systematic bias in these correlations revealed OpenEP to outperform the clinical platform in recovering accurate values. Both activation and voltage mapping data created with OpenEP were correlated with clinical values (mean voltage *R*^2^ = 0.8708, *P* < 0.001; local activation time *R*^2^ = 0.8892, *P* < 0.0001). OpenEP provides the processing necessary for 87 of 92 qualitatively assessed analysis techniques (95%) and 119 of 136 quantitatively assessed analysis techniques (88%) in a contemporary cohort of mapping studies.

**Conclusions:**

We present the OpenEP framework for evaluating electroanatomic mapping data. OpenEP provides the core functionality necessary to conduct electroanatomic mapping research. We demonstrate that OpenEP is both space-efficient and accurately representative of the original data. We show that OpenEP captures the majority of data required for contemporary electroanatomic mapping-based electrophysiology research and propose a roadmap for future development.

## Introduction

Electroanatomic mapping systems are used extensively to guide catheter-based ablation procedures (Kim et al., 2020). Electroanatomic mapping system guided procedures are extremely successful under certain conditions but there is significant variability in outcomes reported (Gaita et al., 2008; Taghji et al., 2018). Despite advancements in the understanding of the pathophysiology of both atrial (Iwasaki et al., 2011; Hansen et al., 2018; Lau et al., 2019) and ventricular arrhythmias (Anter et al., 2016; Pokorney et al., 2016; Aziz et al., 2019), this outcome variability indicates that there is still much to learn about the electropathophysiology of these arrhythmias, how electrical and structural abnormalities can be quantified by electroanatomic mapping systems and how appropriate therapeutic targets can be identified and treated using ablation.

Electroanatomic mapping systems provide several core functions including catheter localization, anatomical representation, electrophysiological map construction, and localization of ablation lesions. As such, the data acquired by these systems provides key information about atrial or ventricular myocardial morphology and electrical function. Such data is interpreted conventionally within electroanatomic mapping platforms through the creation of local activation time maps and their derivatives (Williams et al., 2018), voltage maps (Kistler et al., 2004; Pak et al., 2011; Al-Kaisey et al., 2020; Pappone et al., 2020), and maps representing electrogram morphological features during arrhythmia or pacing (Chang et al., 2013; Jadidi et al., 2016). Within research settings, the same data has also been extensively post-processed to analyze complex electrogram features (Almeida et al., 2020; Vraka et al., 2020), activation patterns (El Haddad et al., 2014), conduction velocities (Cantwell et al., 2015; Aronis et al., 2020) and identify phase singularities through multiple mapping techniques (Child et al., 2018; Ríos-Muñoz et al., 2018).

All of these post-processing steps depend on common data management processes including the ability to export mapping data from clinical systems, store this data in space-efficient machine-readable formats and access electrophysiological data for post-processing. Although multiple research groups are active in these areas, there is as-yet no reported, open-source, standardized framework for performing these core functionalities. The creation of software to achieve these functions represents a barrier to entry to electrophysiology research and the lack of a common data standard represents a hindrance to collaboration between research groups.

We sought to develop a standardized data structure for electroanatomic mapping data together with a framework for parsing data from commonly used electroanatomic mapping platforms to facilitate electroanatomic data processing for research purposes. Here we present the OpenEP (Open Electrophysiology Interface for Research) framework, associated code repositories and website^1^. We further provide examples analyzing electrophysiology data using OpenEP, benchmark its storage efficiency compared to the original raw data and validate performance against the original data.

The three aims of this study were therefore (1) to present an open research data standard for storing and parsing electroanatomic mapping data; (2) to analyze the performance of an implementation framework using this data standard for storing and representing electroanatomic mapping data; and (3) to determine, through literature review, the suitability of OpenEP for contemporary research thereby presenting a roadmap for future development.

## Materials and Methods

### Data Structure and Implementation

The computer code shared within OpenEP has been under continual development for over a decade and is actively used within our research groups to analyze data from the major electroanatomic mapping platforms. This active use permits its ongoing development. The software described here is made available under the Apache License 2.0 and can be freely used for academic research.

Inspection of data exported from Velocity, Precision and Carto3 electroanatomic mapping system revealed two categories of electroanatomic mapping data – surface data and electrogram data. Individual exported datatypes representing all geometric and electrical data acquired by the mapping system were grouped into each of these categories. An etymology was designed categorizing each datatype into subgroups within these categories (see “Supplementary Material”). An implementation of OpenEP was developed using MATLAB R2020a (The MathWorks, Inc.).

### Clinical Data

For the purposes of this evaluation of the OpenEP software, left atrial activation/voltage mapping data was exported from one electroanatomic mapping platform (Carto3; V6). The general format of this data consisted of a series of XML files describing the study characteristics, a series of text files, 12 per mapping point, describing the electrogram features, and a file describing the chamber geometry and electroanatomic maps created during the clinical case. Patient datasets used in this study included forty patients undergoing first-time atrial fibrillation ablation. Example datasets for use with OpenEP are available in the Supplementary Material. Prior to data export from the electroanatomic mapping platform, all electrograms were manually inspected. Electrograms which were clearly far-field were excluded from the electroanatomic maps and timing annotations were corrected as necessary. Anatomical structures were added using the mapping system to represent the mitral valve annulus and all pulmonary vein ostia. Clinical data was collected during routine patient care. Health Research Authority approval was granted for the retrospective use of this data for research (REC Reference: 18/HRA/0083).

### Performance Benchmarking

To benchmark the performance of OpenEP, two metrics were considered. Firstly, the time taken to import the data and create the OpenEP data structure for each dataset was calculated. A recursive script was set up to automate measurement of data import time for each dataset. Performance benchmarking was performed on MacOS (MacBook Pro, 3.3 GHz dual core i7 processor, 16 Gb RAM, 500 Gb SSD storage), using the MATLAB environment (R2020a). Secondly, dataset size for each patient was measured using the standard operating system tools and compared with the dataset size exported by the clinical mapping system in both compressed and uncompressed (zip) formats.

### Data Validation

The OpenEP data format can be used for investigation of the electropathophysiology of both atrial and ventricular arrhythmias. Here we focus on using OpenEP for atrial fibrillation electroanatomic mapping and ablation data. To benchmark the data validity of OpenEP, four analyses were performed.

Firstly, chamber volume and chamber surface area were calculated using OpenEP and compared to chamber volume calculated using the clinical mapping system. Chamber surface area was calculated based on the original mesh including the anatomical structure cut-outs (open) and based on the same mesh with any anatomical structures closed (closed) using the OpenEP functions. As an example, for the left atrium the “open” mesh is a mesh with cut-outs in place for the mitral valve and pulmonary veins whilst a “closed” mesh is a mesh with each of these anatomical structures filled in.

Secondly, the performance of OpenEP for reproducing electroanatomic maps was considered. OpenEP provides functions to display electroanatomic maps created by the clinical system as well as additional functions to re-create electroanatomic maps from raw electrogram data. To validate these functions the total activation time (TAT) and site of earliest activation were calculated for both classes of local activation time maps, and the mean chamber voltage and percentage are of low voltage were calculated for both classes of voltage maps.

Thirdly, the number of electroanatomic mapping data points identified by OpenEP was compared to the expected number of electroanatomic mapping data points based on the clinical system to ensure that all mapping points were correctly identified and parsed.

Finally, the number and position of ablation points was compared between OpenEP and the clinical systems.

### Literature Survey

To determine the “real world” requirements for an electrophysiology research data storage format we performed a literature search using PubMed^2^ for the following terms: “(electroanatomic mapping) AND [(atrial fibrillation) OR (ventricular tachycardia)]”. To ensure applicability to contemporary research the search was limited to the previous 1-year (November 2019–2020, see “Supplementary Material”). Abstracts were screened to identify research studies in which data export of clinical electroanatomic mapping data was required. Review articles, case reports and case series using standard electroanatomic mapping techniques to deliver clinical treatments were excluded. Full text review was performed to identify data types that were analyzed for the purposes of these studies. The following 19 data types, exposed through OpenEP APIs were tabulated: chamber geometry, number of mapping points, location only points, anatomical structures, electrogram locations, bipolar electrograms, unipolar electrograms, contact force, ablation positions, ablation temperature, ablation power, ablation time, and ablation lesion indices (e.g., ablation index or lesion size index), impedance, local activation time annotations, local activation time map, bipolar map, unipolar map and fractionation map. For each study, each data type was given a score from 0–4 with 0 = data type not used; 1 = qualitative analysis using the clinical system; 2 = quantitative analysis using the clinical system; 3 = qualitative analysis following data export; and 4 = quantitative analysis following data export. Additional data types, not available through OpenEP were also considered. The frequency of occurrence of each of these data types was calculated. The percentage of studies for which OpenEP would have provided complete input data was subsequently calculated.

## Results

### Implementation

**FIGURE 1:**
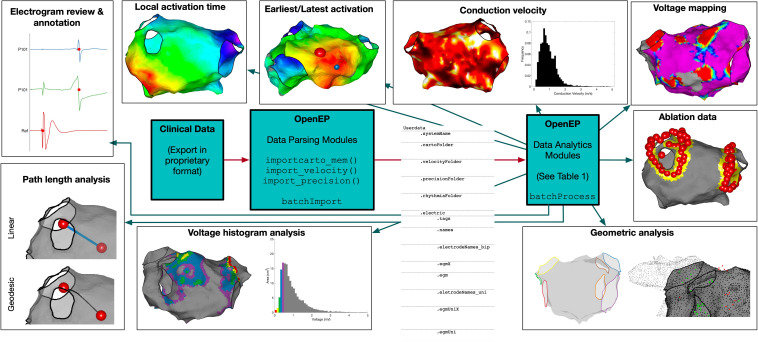
OpenEP Overview. The core components of OpenEP are the data parsing modules (used to parse data from proprietary clinical system formats) and the data analytics modules (used to access and analyze the data stored in OpenEP format).

On overview of the OpenEP architecture is shown in Figure 1. The basic architecture consists of the OpenEP data format, together with Data Parsing modules and Data Analytics modules. Implementations of the OpenEP standard have been created for three clinical systems to date: Carto3 (Biosense Webster), Velocity (St Jude Medical) and Precision (Abbot). Following data export from one of these systems, processing a dataset using OpenEP begins with a call to an import function, for example, importcarto_mem(), importprecision(), or importvelocity(). Calling these functions from the command window without arguments prompts the user to perform selections to identify the study files, the clinical map of interest, the reference mapping channel and an ECG channel. All of these selections can also be passed as arguments to importcarto_mem() to allow command line-only interaction. Subsequent parsing of the dataset is entirely automated and results in the creation of a data structure called userdata in the workspace, which can also be saved to disk. A full description of each field within this structure is given in the Supplementary Material.

To perform data analysis on multiple patient datasets, two template functions are provided, batchImport() and batchProcess(). The import function takes the same arguments as importcarto_mem() to fully automate the import of multiple patient datasets into OpenEP format. The process function takes as its only argument the absolute path to a directory of OpenEP data files and provides a template for performing data processing sequentially on each dataset before returning the outputs in a structure.

A list of currently available data processing functions is given in Supplementary Table 3, and a live version of the OpenEP code documentation will be hosted online^3^.

### Performance Benchmarking

The clinical datasets consisted of left atrial electroanatomic mapping data created to facilitate atrial radiofrequency ablation for the treatment of atrial fibrillation. There were 963 ± 430 bipolar mapping points per patient (range 209 – 2031 points per map). The data was exported from the clinical mapping system as a single compressed archive, one per patient, containing plane text and XML files. There were averages of 35,175 ± 18,861 text files and 3,177 ± 1,719 XML files, per patient.

**FIGURE 2:**
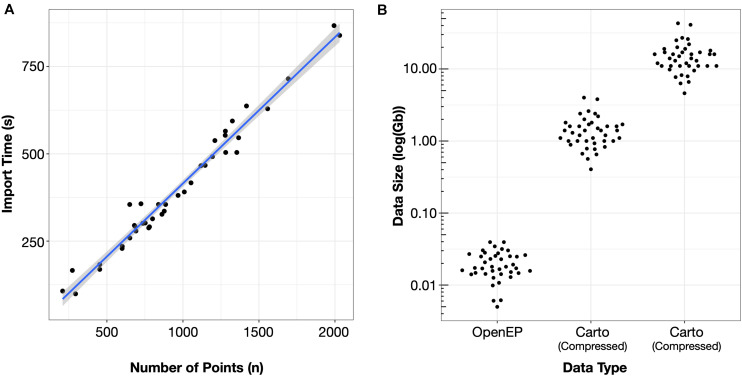
**(A)** Import time was proportional to the number of mapping points in the clinical dataset. **(B)** Storage space required for electroanatomic mapping data was three orders of magnitude smaller than the uncompressed Carto data and two orders of magnitude less than the compressed Carto data.

The time taken per case to import the electroanatomic mapping data was 400 ± 162 s. The time taken to import the datasets was significantly correlated with the number of mapping points in the dataset (*R*^2^ = 0.9719, *P* < 0.0001) (Figure 2A).

The mean OpenEP dataset size was 20.5 ± 8.7 Mb, which was significantly smaller than both the compressed (1.46 ± 0.77 Gb) and uncompressed (15.54 ± 8.08 Gb) export files from the electroanatomic mapping system (Figure 2B).

### Data Validation

#### Chamber Geometry

**FIGURE 3:**
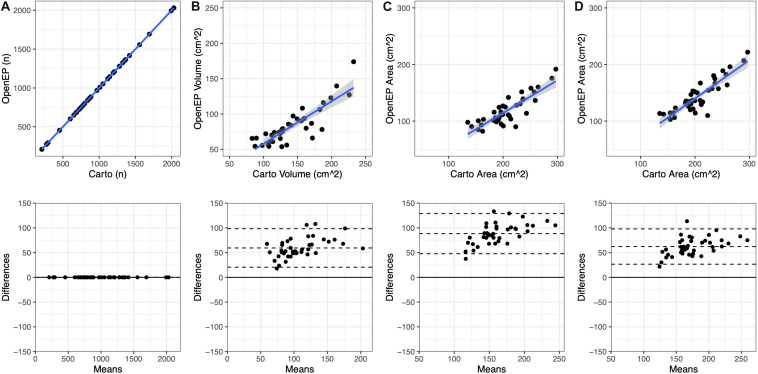
Geometric measurements compared between the original electroanatomic mapping system and OpenEP. **(A)** Number of mapping points present in the original Carto map and subsequently identified by OpenEP, using getNumPts(userdata). **(B)** Chamber volume measured by Carto and OopenEP, using getVolume(userdata). **(C)** Chamber area measured by Carto and OpenEP, using getArea(usredata, 'method', 'nofill'). **(D)** Chamber area measured by Carto and OpenEP, using getArea(usredata, 'method', 'fill').

**FIGURE 4:**
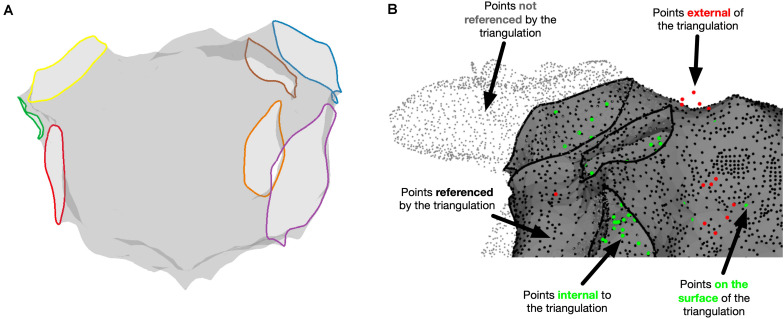
Miscellaneous OpenEP functions. **(A)** Identification of anatomical structures using the OpenEP command: getAnatomicalStructures(userdata, 'plot', true). **(B)** Identification of point status for points referenced in userdata using [inoutpts, meshrefpts] = pointStatus(userdata, 'plot', true).

The relationship between chamber geometry metrics measured using the electroanatomic mapping platform and OpenEP is shown in Figure 3. There was an excellent correlation between Carto-derived metrics and OpenEP-derived metrics for chamber surface area “open” (*R*^2^ = 0.7187, *P* < 0.0001) and “closed” (*R*^2^ = 0.7726, *P* < 0.0001). There was a moderate correlation between Carto-derived metrics and OpenEP-derived metrics for chamber volume (*R*^2^ = 0.5179, *P* < 0.0001). Visual inspection of Bland-Altman plots showed that there was both systematic and proportional bias in the measurement of all three metrics metric, which was confirmed by weak but significant linear regression analysis of all three plots (Area, open *R*^2^ = 0.349, *P* < 0.0001; Area, closed *R*^2^ = 0.2204, *P* = 0.0013; Volume *R*^2^ = 0.2551, *P* = 0.0009). Functions are available within OpenEP to visualize chamber geometry, anatomical structures, and provide information about vertices within the geometry (Figure 4).

#### Local Activation Time Mapping

**FIGURE 5:**
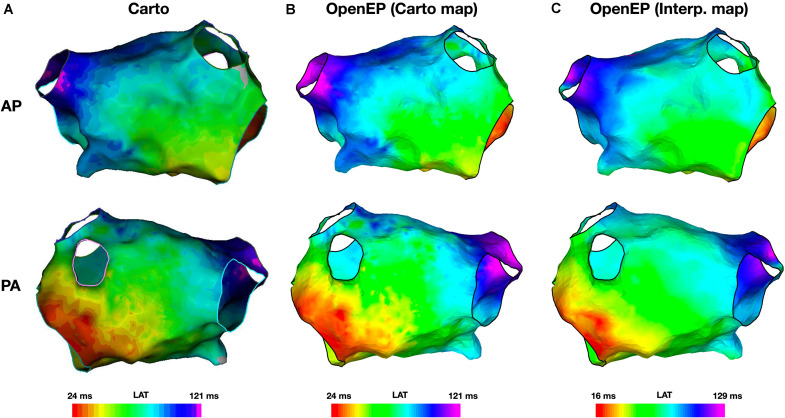
Local activation time mapping. **(A)** Activation maps exported from the Carto electroanatomic mapping platform. **(B)** Activation map created by OpenEP using the Carto electroanatomic mapping data. OpenEP command: drawMap(userdata, 'type', 'act', 'orientation', 'ap'). **(C)** Activation map created by OpenEP using the Carto electrogram data. OpenEP command: interpData = generateInterpData(userdata, 'lat-map'); drawMap(userdata, 'type', 'act', 'orientation', 'ap', 'data', interpData). AP, antero-posterior; PA, postero-anterior; LAT, local activation time.

Example local activation time maps created using Carto and using the interpolation functions built into OpenEP are shown in Figure 5. Local activation time maps were quantified using TAT, the site of earliest activation and by a point-by-point comparison of activation times.

**FIGURE 6:**
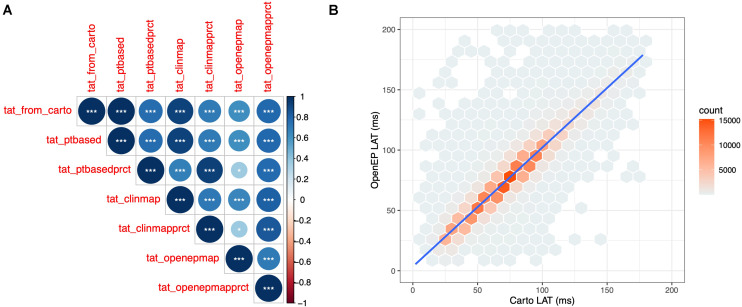
Quantification of OpenEP local activation time maps. **(A)** Cross correlation matrix comparing Carto-defined total activation time with the six total activation time metrics available in OpenEP. **(B)** Point-by-point comparison of Carto-defined local activation time and OpenEP-defined local activation time maps.

The total activation time was defined as the difference in activation times between the earliest and latest activation time mapping points on the Carto system. OpenEP can recover this metric from the exported data (“Point-based TAT”) and provides five additional metrics for calculating total activation time as described in Supplementary Table 1. TAT was calculated for all 40 patient datasets, using all six methods and compared with Carto-derived total activation time. There was a perfect correlation between Carto-derived TAT and OpenEP point-based TAT (*R*^2^ = 1, *P* < 0.001). In the era of high ultra-high density mapping these point-based metrics are vulnerable to annotation errors and therefore map-based and percentile-based methods are also provided. The correlations between these methods are shown in Figure 6A.

**FIGURE 7:**
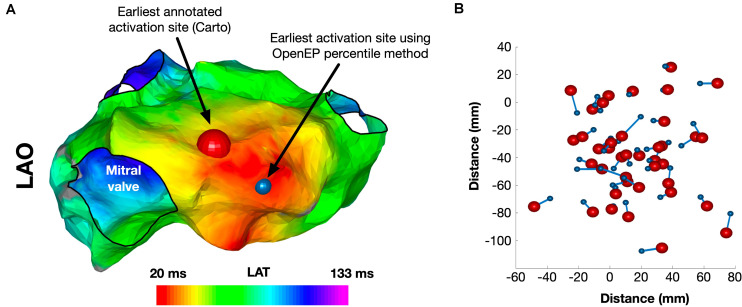
Identification of the site of earliest activation. **(A)** Example activation map showing the site of earliest activation, defined as the earliest local activation time recorded by Carto shown in red, and the site of earliest activation using the OpenEP percentile method shown in blue. **(B)** Relationship between Carto earliest activation sites and OpenEP earliest activation sites for all 40 cases. The Carto-defined earliest activation sites are shown with red spheres and the OpenEP-defined earliest activation sites are shown with blue spheres. The connecting lines indicate the pairing of data points on a case-by-case basis. LAO, left anterior oblique; LAT, local activation time.

The site of earliest activation was defined as the earliest point identified on the Carto-defined local activation time map. Again, OpenEP can recover this position but provides alternative methods to compute the earliest activation point, analogous to the methods for total activation time shown in Supplementary Table 2. A comparison of Carto-defined earliest activation and the percentile-based electrogram method (“ptbasedprct”) is shown in Figure 7A for a single case and summarized in Figure 7B for all 40 cases in the validation dataset. The mean distance between Carto-defined and OpenEP-defined earliest activation points was 10.8 ± 4.4 mm.

A point-by-point comparison of all surface based local activation times was performed. The point-by-point comparison of Carto-derived and OpenEP-derived interpolated local activation time maps revealed a highly significant correlation between these two metrics (*R*^2^ = 0.8892, *P* < 0.0001) (Figure 6B).

**FIGURE 8:**
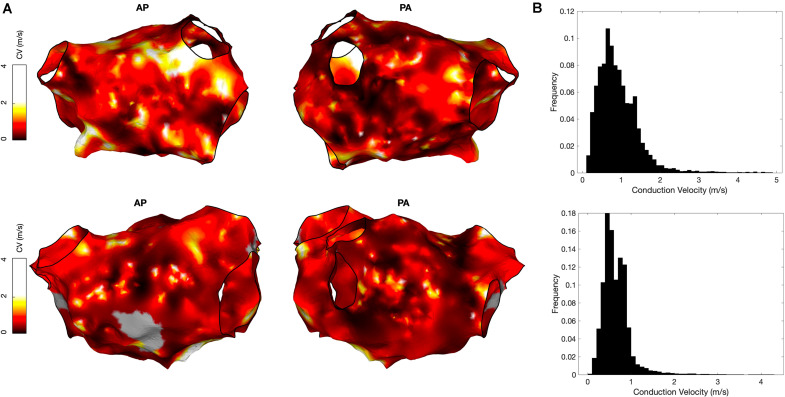
Conduction velocity measurement using OpenEP software. **(A)** Conduction velocity maps of two cases in anterior-posterior orientation (left) and postero-anterior orientation (right). Maps created using the OpenEP function: drawMap(userdata, 'type', 'cv', 'coloraxis', [0 2], 'orientation', 'pa'). **(B)** Conduction velocity histograms corresponding to the maps in panel A; created using the OpenEP function call: cvHistogram(userdata). CV, conduction velocity; AP, antero-posterior; PA, postero-anterior.

OpenEP also includes functions to create conduction velocity maps from local activation time maps, which can be displayed using the drawMap.m function. In addition, conduction velocity histogram analysis is available via the cvHistogram.m function (Figure 8). Currently, OpenEP provides a single method to calculate conduction velocity which uses the radial basis function method (Masè and Ravelli, 2010).

#### Voltage Mapping

**FIGURE 9:**
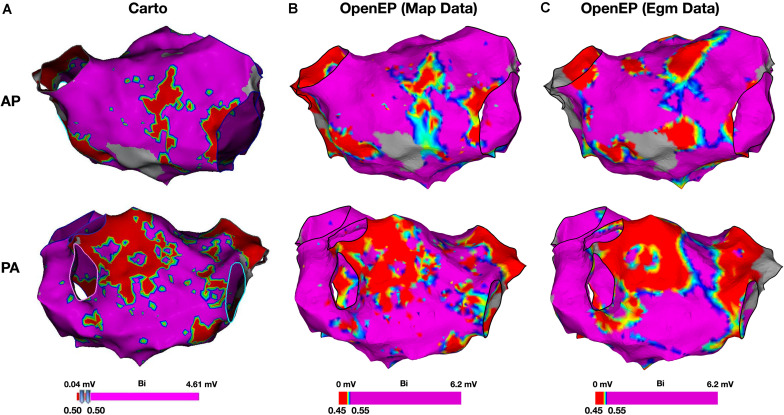
Bipolar voltage mapping. **(A)** Voltage maps created using Carto, with a voltage threshold of 0.5 mV. **(B)** Voltage maps created using OpenEP with a voltage threshold range of 0.4–0.6 mV applied to the voltage mapping data exported by Carto. OpenEP command: drawMap(userdata, 'type', 'bip', 'coloraxis', [0.4 0.6], 'orientation', 'pa', 'colorfillthreshold', 10); **(C)** Voltage maps created using OpenEP with a voltage threshold range of 0.45–0.55 mV, interpolated from the raw electrogram data at every mapping point and with a color fill threshold of 10 mm. OpenEP command: interpBip = generateInterpData(userdata, 'bip-map'); drawMap(userdata, 'data', interpBip, 'type', 'bip', 'coloraxis', [0.4 0.6], 'orientation', 'pa'). AP, antero-posterior; PA, postero-anterior; Bi, bipolar voltage.

**FIGURE 10:**
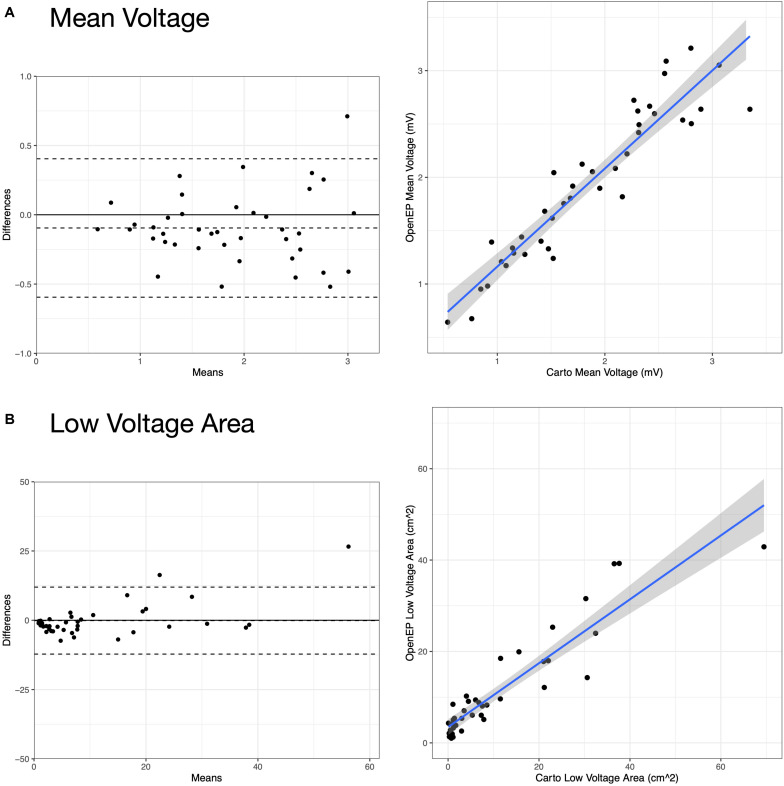
Analysis of Carto and OpenEP voltage mapping data. **(A)** Assessment of mean chamber voltage using Carto and OpenEP. **(B)** Assessment of low voltage area using Carto and OpenEP. The OpenEP commands: getMeanVoltage(userdata, 'method', 'map'); getMeanVoltage(userdata, 'method', 'egm'); getLowVoltageArea(userdata, 'method', 'map'); and getLowVoltageArea(userdata, 'method', 'egm') were used to create the data for these figures.

Example voltage maps created directly using Carto and indirectly using the interpolation functions built into OpenEP are shown in Figure 9. Voltage maps were quantified using the mean chamber voltage and the percentage of low voltage (defined as interpolated voltage <0.5 mV). Mean chamber voltage was significantly correlated between Carto and OpenEP voltage maps (*R*^2^ = 0.8708, *P* < 0.001). Similarly, low voltage area defined as the atrial area with voltage less than 0.5 mV was significantly correlated between Carto and OpenEP voltage maps (*R*^2^ = 0.8481, *P* < 0.0001). Scatter plots with regression lines and Bland-Altman plots for the comparison of both metrics are shown in Figure 10.

**FIGURE 11:**
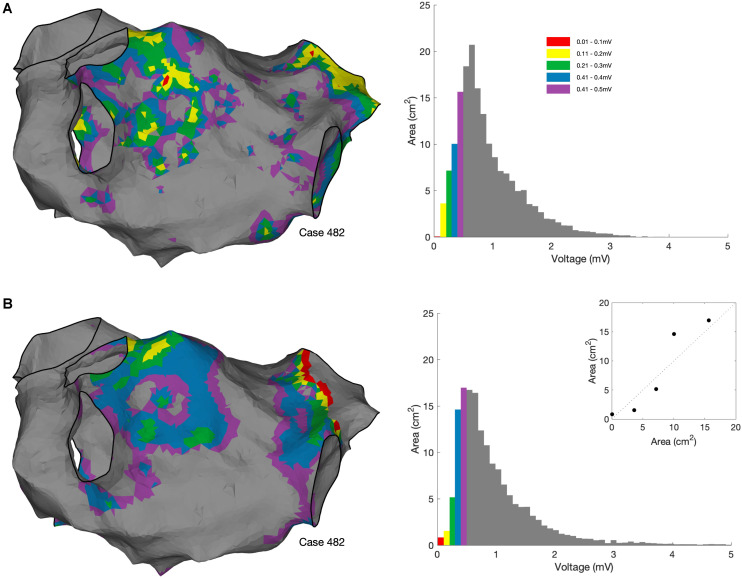
Voltage histogram analysis. **(A)** Voltage histogram analysis performed using bipolar voltages exported from the clinical mapping system. OpenEP command: voltageHistogramAnalysis(userdata, 'plot', true, 'method', 'map'). **(B)** Voltage histogram analysis performed using bipolar voltages re-interpolated from the exported electrogram voltage annotations using the OpenEP command voltageHistogramAnalysis (userdata, 'plot', true, 'method', 'egm'). Inset in lower panel shows the comparison in areas between the two methods.

OpenEP also allows more advanced quantification of voltage metrics including voltage histogram analysis (Figure 11).

#### Electrogram Display

**FIGURE 12:**
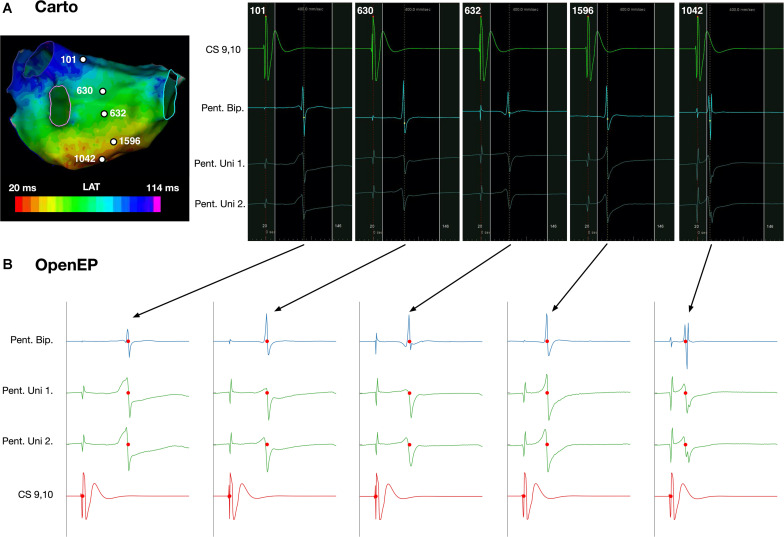
Display of electrogram data using OpenEP. **(A)** Reference, bipolar and unipolar electrogram data at five selected sites on the posterior wall of a left atrium. **(B)** Corresponding electrograms extracted and plotted using OpenEP. Blue – bipolar electrogram; green – paired unipolar electrograms; red – reference coronary sinus electrogram. Red dots indicate the activation time annotations extracted from the clinical mapping platform. OpenEP function example: plotOpenEPEgms[userdata, 'iegm', getIndexFromCartoPointNumber(userdata,1042)]. LAT, local activation time; CS, coronary sinus; Pent, PentaRay; Uni, Unipole; Bip, Bipole.

OpenEP can be used to simplify the process of accessing electrograms from electroanatomic mapping data. For Carto data, the functions getIndexfromCartoPointNumber() and plotOpenEPEgms() are provided which can be used together to plot a figure containing the electrogram pertaining to a specific electroanatomic mapping point. Examples of such electrograms and comparison with the clinical electrograms are shown in Figure 12. The OpenEP function, plotOpenEPEgms() accepts a number of parameter/value inputs to customize the output which are summarized in Supplementary Table 2.

#### Ablation Point Input and Display

**FIGURE 13:**
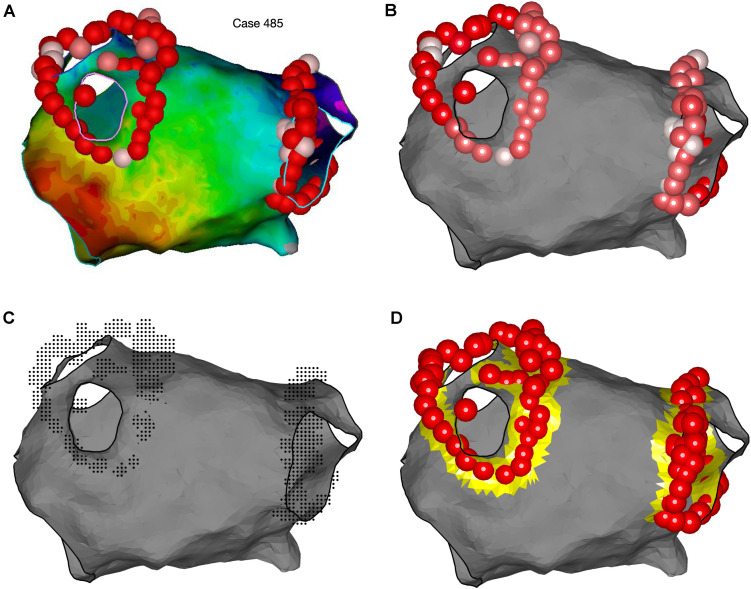
Representation of ablation points and quantification of ablation area using OpenEP. **(A)** Ablation lesion representation in the Carto electroanatomic mapping platform. **(B)** Ablation lesion representation using OpenEP. Ablation lesions are colored according to the Ablation Index (low = white; high = red). OpenEP function call: plotVisitags(userdata, 'color', visitag.tag.index.value). **(C)** Specifically for the Carto electroanatomic mapping platform the “grid” of ablation positions is also exposed together with all ablation-related data (impedance, time, temperature, and contact force). OpenEP function call: plotVisitags(userdata, 'plot', 'grid'). **(D)** Ablation area can be calculated with the OpenEP function call: ablArea = getAblationArea (userdata). Ablation area can be added to an existing plot using the OpenEP function: plotAblationArea(userdata).

OpenEP offers two tools that can be used for identifying ablation sites. Firstly, ablation sites may be tagged within location-only points. These points are labeled as such in userdata.electric.tags and have location data stored in userdata.electric.egmX and .egmSurfX but have no linked electrical data. Modern electroanatomic mapping systems provide metrics which quantify energy delivery (and seek to predict lesion size) during radiofrequency ablation, such as the Lesion Size Index (Whitaker et al., 2018) and Ablation Index (O’Neill et al., 2019). Since these indices vary per-platform and per-case, OpenEP provides helper functions for accessing radiofrequency index data which is appended to userdata and then stored in the subfields of userdata.rfindex. So far, only Visitags (Carto3) are implemented via the importvisitag() function but the roadmap for development prioritizes the parsing of Lesion Stability Index (Precision). Example data is shown in Figure 13, and the format of the dataset created is shown in the Supplementary Material. Additional functions are provided to plot the ablation sites, colored by any available ablation parameter and calculate ablation area. Ablation parameters (time, force, impedance, temperature, and power) can currently be plotted from the available raw data and the roadmap for development includes the provision of help functions to streamline these graphing functions.

### Literature Survey

Following the initial literature search, 136 suitable articles were identified (see “Supplementary Material”). Case reports (*n* = 18), clinical series using electroanatomic mapping for treatment (*n* = 5), editorials (*n* = 5), guidelines (*n* = 6), conference abstracts (*n* = 1), non-electroanatomic mapping studies (*n* = 30), non-English language studies (*n* = 4) and review articles (*n* = 10) were excluded leaving 46 studies for analysis.

**FIGURE 14:**
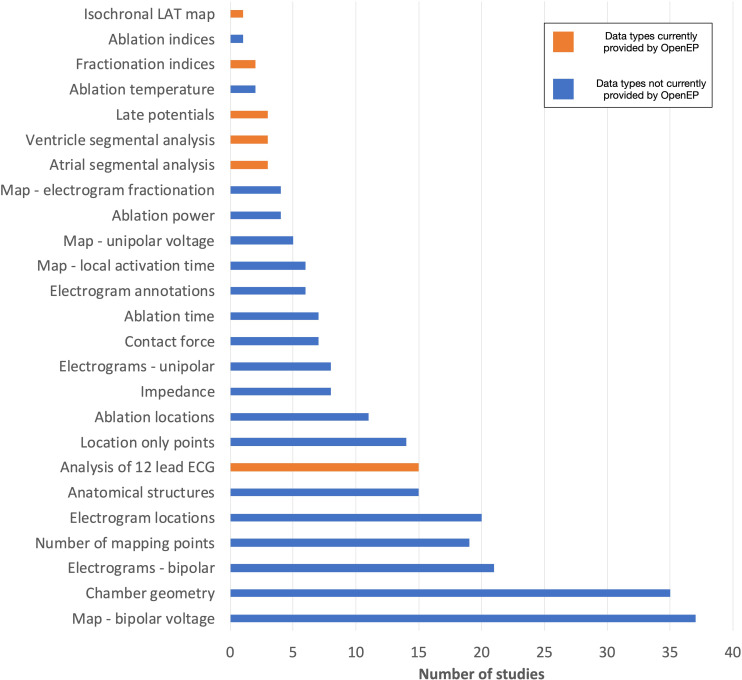
Data types assessed by contemporary electroanatomic mapping studies. Blue bars represent the data types currently accessible through OpenEP. Orange bars represent the data types which are not currently accessible through OpenEP but which form objectives in the Roadmap for Development (https://openep.io/roadmap).

The frequency of data types analyzed amongst all the studies is shown in Figure 14.

Studies were scored according to the highest level of data analysis performed, ranging from qualitative analysis on the clinical system (score = 1) to quantitative analysis following data export (score = 4). Of the included studies, 6/46 (13%) performed qualitative analysis on the clinical system and 30/46 (65%) performed quantitative analysis on the clinical system. A minority of studies (10/46, 22%) performed data export from the clinical system, and all of these studies performed quantitative analysis of at least some electroanatomic mapping data. Of all the studies analyzed, 21/41 (51%) performed quantitative analysis of chamber geometry or low voltage areas manually using area measurement tools embedded in the clinical system.

The current implementation of OpenEP exposes access to the full electroanatomic mapping dataset and analysis techniques required for completion of 31/46 studies (67%). When image integration and registration-type analyses, for which there are several existing software platforms, are excluded this figure rises to 36/46 (78%). Additional electroanatomic mapping data requirements included access to full 12-lead ECGs at each mapping point (4 studies), re-calculation of electrogram complexity/fractionation indices (2 studies), analysis of late potentials (3 studies), segmental analysis of the atria (3 studies) or ventricles (3 study) and creation of isochronal local activation time maps (1 study). In addition, image integration analysis – for example registering electroanatomic mapping data to imaging data, importing imaging data into a clinical system or exporting imaging data from a clinical system – was performed in 7 studies.

Considering all the analysis techniques applied across all the studies together, 101 analysis techniques were performed qualitatively on the clinical system, 112 analysis techniques were performed quantitatively on the clinical system, 2 analysis technique was applied qualitatively following data export and 41 analysis techniques were performed quantitatively following data export. In summary qualitative analysis was performed for 103 analysis techniques and quantitative analysis was performed for 153 analysis techniques. Considering each class of analysis technique (qualitative vs. quantitative) separately, the current OpenEP framework would have provided access to 96 of 103 qualitatively assessed data points (93%) and 134 of 154 quantitatively assessed data points (87%). In doing so, OpenEP removes a barrier to clinical electrophysiology research and facilitates offline analysis of electrophysiology data.

## Discussion

In this study we introduce the OpenEP (Open Electrophysiology Interface for Research) framework and provide performance and validation benchmarking. We demonstrate improvements in data storage efficiency for clinical electroanatomic mapping data. We illustrate the simplicity of using OpenEP for data analysis activities in electrophysiology research, many of which can be executed using single-line function calls. We further demonstrate, through a retrospective assessment of recent literature, the suitability of the OpenEP data format for representing electroanatomic mapping data used in contemporary arrhythmia research. Finally, we introduce the OpenEP website^4^ which will provide code documentation, example datasets and outlines the roadmap for future development. All source code referred to in this work is linked to from the OpenEP website and is licensed under the Apache License 2.0. The release used in this paper is archived with Zenodo (DOI: 10.5281/zenodo.4471319 and available from https://doi.org/10.5281/zenodo.4471319 (Williams and Linton, 2021).

A key advantage of the proposed framework for data analysis is that the methods and algorithms are published in full, allowing inspection by collaborators, other researchers or industrial partners. In particular this development can ensure confidence in the published methods. Notably, the literature survey performed here identified that a majority of recent electroanatomic mapping studies performed area measurements of either an entire chamber or of specific regions (for example low voltage regions). However analysis using OpenEP showed that there were both systematic and proportional biases in the assessment of chamber area. Consistent with this observation are the existing reports that manual measurements of low voltage areas are error prone (Herczeg et al., 2020a,b). In contrast, area measurements in OpenEP are implemented using conventional geometric techniques. Whilst every effort has been taken to ensure their correct implementation, the open nature of the platform further allows others to confirm the accuracy of these implementations for themselves. Finally, by providing a standard analysis method which can be used by any researchers in future studies the provision of this platform could ensure comparability between such studies.

Minimizing data storage requirements is a further benefit of the OpenEP framework. There are three ways in which the OpenEP format improves data storage requirements. Firstly, OpenEP eliminates redundancy in the data such that there is only one copy of every unique electrogram. Secondly, the entire dataset (including anatomical and electrical data) is stored as a single data structure rather than multiple individual files which eliminates the file system overheads necessary to store large numbers of files. Finally, the data is stored as a binary file rather than a series of text files. In the format exported by the clinical mapping systems each individual patient data set is typically in the order of 1–2 Gb in size. The OpenEP format significantly reduced the storage requirements for this dataset. Given that typical electroanatomic mapping studies may recruit 1–2 hundred patients it is not uncommon for the data storage requirements for one study to be greater than that available on a single personal computer. Furthermore, transferring data between external storage media for access is time consuming, especially when many thousands of individual files make up one patient dataset. Aside from the convenience aspect of improved data storage there is increasing awareness of the environmental impact of wasteful data storage practices (Lucivero, 2020). In this context, the OpenEP framework allows electroanatomic mapping data to data to be stored in an efficient manner.

We also highlight that the OpenEP data structure has been designed with extensibility in mind, most easily illustrated with an example. When creating geometric maps of electrophysiological parameters – such as electrogram voltage or activation time – a three-dimensional interpolation is necessary to create a visual color representation of the physiological parameter of interested. This interpolation is commonly performed using commercially available clinical electroanatomic mapping platforms. OpenEP permits access to, and analysis of, these clinical data interpolations. However, there are numerous methods to perform spatial interpolation which can result in different interpretations of the same data. OpenEP therefore provides its own internal framework for performing interpolations based on the originally acquired electrical data. The OpenEP function generateInterpData() is a key function for carrying out this task and can be easily modified/extended to make use of alternative methods for data interpolation. A further example of the extensibility of the OpenEP data structure is in the visualization routines. These routines make use of data “getter” methods which access the required data from the OpenEP data structure. For example, plotOpenEPEgm() makes use of getOpenEPEgm() and quantifyVoltageDistribution() makes use of getVoltageDistribtion(). By separating the visualization routines from the “getter” routines it is possible to easily implement alternative visualization techniques whilst making use of the same data as the OpenEP framework.

As noted above the OpenEP framework has been in active development and use for over a decade within our own research groups (Linton et al., 2009; Jamil-Copley et al., 2013; Williams et al., 2017, 2018, 2019; Whitaker et al., 2018). As such it has evolved, project by project, to include additional functionality when required. In order to evaluate how well this functionality now maps to functionality required in contemporary electroanatomic mapping studies a literature review was performed to assess the datatypes and analysis methods in use in the previous 1 year of electroanatomic mapping studies (November 2019–2020, see “Supplementary Material”). This analysis revealed that the majority of data types required for recent studies are now exposed through OpenEP functions. This analysis also revealed a number of areas for future development including parsing and analyzing full 12-lead ECG signals, providing methods to perform fractionation analysis of intracardiac electrograms, methods to perform segmental analysis of the atria and ventricles and methods to assess late potentials in ventricular tachycardia studies. These areas have now been mapped to the roadmap for future development, which will be made available through the OpenEP website^5^.

One area that is included in the roadmap for future development is the implementation of alternative methods for calculating conduction velocity. Although a simple concept, the measurement of conduction velocities from clinical data is challenging with multiple previous techniques proposed including triangulation of electrode positions/activation times (Kojodjojo et al., 2006a,b, 2007; Sawa et al., 2008; Ravelli et al., 2011; Cantwell et al., 2014), vector loops and omnipole mapping (Kadish et al., 2003; Massé et al., 2016; Deno et al., 2017), cosine-fit techniques (Weber et al., 2011; Roney et al., 2014, 2019) polynomial fit techniques (Nalliah et al., 2021) and calculation of the spatial gradients of local activation fields (Mourad and Nash, 2007). The method currently implemented in OpenEP uses radial basis function interpolation (Masè and Ravelli, 2010). Future work is planned to incorporate other conduction velocity measurement techniques within the OpenEP framework.

Related to conduction velocity is the concept of local activation time assignment. Currently, local activation time assignment within OpenEP is taken from the clinical mapping system. However, it could be useful to perform activation time assignment within OpenEP itself in order to create activation maps which are agnostic to the clinical system used for collecting electrogram data. Several OpenEP functions including getElectrogramX(…), getEgmsAtPoints(…), and getWindowOfInterest(…) will be particularly useful for developing local activation time assignment functionality which is not yet part of OpenEP.

During the literature review process we identified two prior studies (Brett et al., 2020; Hohmann et al., 2020) that have made code available for accessing electroanatomic mapping data. In these study the system-created voltage maps alone were exported from clinical systems and a parser was written to import these data into the 3D Slicer program. These computer codes do not therefore allow access to the full array of electroanatomic mapping/ablation data exposed by OpenEP. Compared to this study the OpenEP framework provides access to all the individual datatypes available from the electroanatomic mapping platforms including raw electrogram data, ECG data, ablation data and interpolated electrophysiological maps and further provides methods to visualize and analyze mapping, electrogram and ablation data. In addition, through these series of analyses we have benchmarked and validated the current performance of the OpenEP framework and provided a roadmap for its future development.

### Limitations

The OpenEP framework will likely never be in a position where it could be considered “complete.” Indeed, electroanatomic mapping platforms are evolving all the time and the OpenEP framework will need to continually evolve in order to continue to represent contemporary data. However, we hope that by making the software available under an open-source license we will encourage other researchers to become actively involved in this development process and we welcome them to do so.

Based on our experience during the years of developing this framework, this code is entirely based on the Matlab software. This is a limitation which necessitates access to a Matlab executable in order to run the code. Whilst many researchers will have access to Matlab through their institution, this is not ubiquitous and may limit use of the code. One proposal within the roadmap for development is to create a standalone version of the platform which can be used with only the Matlab runtime environment which does not require a license to access whilst a further development could modify the OpenEP framework to be able to use the open-source Octave platform. A more extensive refactoring to use Python, instead, would be more involved but may lead to advantages in terms of usability and extensibility and is under active consideration.

The literature review performed here highlighted a number of additional functionalities that may be useful for certain contemporary studies. Amongst these we have prioritized segmental analysis of the atria and ventricles as key targets and included these within the roadmap for development. However, to complete segmental analysis will currently require code functionality that is not currently available within OpenEP and will need to be developed.

The opportunity exists to improve the visualization functions within OpenEP. For example, the rendering of local activation time maps using the drawMap.m function has currently been implemented to closely resemble the maps created by the clinical electroanatomic mapping systems, using a modification of the rainbow color map. However, it is recognized that the rainbow color map has several limitations (Borland and Taylor, 2007). Improvements such as rendering isochronal lines could improve the representation of continuous scale data such as local activation times. This objective has been included in the Roadmap for Development.

## Conclusion

In conclusion here we present the OpenEP framework for electrophysiology research, demonstrate its space-efficiency, benchmark its performance and validate the data exposed by the framework. By making the source code available to the research community along with a supporting website we hope that the OpenEP framework can provide the simultaneous benefits of lowering the barriers to conducting contemporary electrophysiology research whilst standardizing the approach to many of the core data processing functions required to conduct such research.

## Data Availability Statement

The original contributions presented in the study are included in the article/Supplementary Material, further inquiries can be directed to the corresponding author/s.

## Ethics Statement

The studies involving human participants were reviewed and approved by the Health Research Authority; 18/HRA/0083. Written informed consent for participation was not required for this study in accordance with the national legislation and the institutional requirements.

## Author Contributions

SW and NL conceived and designed the analysis, wrote computer code, performed the analysis, and wrote the manuscript. CR and CC contributed to the analysis, development of the code, and reviewed and approved the manuscript. JW tested the computer code, collected the data from clinical systems and, reviewed and approved the manuscript. IS, DO’H, IK, and LO’N collected the data from clinical systems, performed preparation of the clinical data, and reviewed and approved the manuscript. MW and MO’N performed clinical data collection during clinical procedures and reviewed and approved the manuscript. MB and SN critically analyzed the results, contributed to analysis designed, and reviewed and approved the manuscript. All authors contributed to the article and approved the submitted version.

## Conflict of Interest

The authors declare that the research was conducted in the absence of any commercial or financial relationships that could be construed as a potential conflict of interest.

## Footnotes

1 http://openep.io

2 https://pubmed.ncbi.nlm.nih.gov

3 http://openep.io

4 http://openep.io

5 http://openep.io

## References

[B1] Al-Kaisey A. M. Parameswaran R. Joseph S. A. Kistler P. M. Morton J. B. Kalman J. M. (2020). Extensive right atrial free wall low-voltage zone as the substrate for atrial fibrillation: successful ablation by scar homogenization. *Europace* 3:euaa233. 10.1093/europace/euaa233 33141888

[B2] Almeida T. Soriano D. Mase M. Ravelli F. Bezerra A. Li X. (2020). Unsupervised classification of atrial electrograms for electroanatomic mapping of human persistent atrial fibrillation. *IEEE Trans. Biomed. Eng.* 10.1109/TBME.2020.3021480 [Epub ahead of print]. 32881680

[B3] Anter E. Tschabrunn C. M. Buxton A. E. Josephson M. E. (2016). High-resolution mapping of postinfarction reentrant ventricular tachycardia: electrophysiological characterization of the circuit. *Circulation* 134 314–327. 10.1161/CIRCULATIONAHA.116.021955 27440005 PMC5072375

[B4] Aronis K. N. Ali R. L. Prakosa A. Ashikaga H. Berger R. D. Hakim J. B. (2020). Accurate conduction velocity maps and their association with scar distribution on magnetic resonance imaging in patients with postinfarction ventricular tachycardias. *Circ. Arrhythmia Electrophysiol.* 13:e007792. 10.1161/CIRCEP.119.007792 PMC7196439 32191131

[B5] Aziz Z. Shatz D. Raiman M. Upadhyay G. A. Beaser A. D. Besser S. A. (2019). Targeted ablation of ventricular tachycardia guided by wavefront discontinuities during sinus rhythm: a new functional substrate mapping strategy. *Circulation* 140 1383–1397. 10.1161/CIRCULATIONAHA.119.042423 31533463

[B6] Borland D. Taylor R. M. (2007). Rainbow color map (still) considered harmful. *IEEE Comput. Graph. Appl.* 27 14–17. 10.1109/MCG.2007.323435 17388198

[B7] Brett C. L. Cook J. A. Aboud A. A. Karim R. Shinohara E. T. Stevenson W. G. (2020). Novel workflow for conversion of catheter-based electroanatomic mapping to DICOM imaging for noninvasive radioablation of ventricular tachycardia. *Pract. Radiat. Oncol.* 11 84–88. 10.1016/j.prro.2020.04.006 32416269

[B8] Cantwell C. D. Roney C. H. Ali R. L. Qureshi N. A. Lim P. B. Peters N. S. (2014). “A software platform for the comparative analysis of electroanatomic and imaging data including conduction velocity mapping,” in *In Proceedings of the 2014 36th Annual International Conference IEEE Engering Medical Biology Society (EMBC)*, (Piscataway, NJ: IEEE). 10.1109/EMBC.2014.6943908 25570276

[B9] Cantwell C. D. Roney C. H. Ng F. S. Siggers J. H. Sherwin S. J. Peters N. S. (2015). Techniques for automated local activation time annotation and conduction velocity estimation in cardiac mapping. *Comput. Biol. Med.* 65 229–242. 10.1016/j.compbiomed.2015.04.027 25978869 PMC4593301

[B10] Chang S. L. Chen Y. C. Hsu C. P. Kao Y. H. Lin Y. K. Lin Y. J. (2013). Electrophysiological characteristics of complex fractionated electrograms and high frequency activity in atrial fibrillation. *Int. J. Cardiol.* 168 2289–2299. 10.1016/j.ijcard.2013.01.194 23465221

[B11] Child N. Clayton R. H. Roney C. H. Laughner J. I. Shuros A. Neuzil P. (2018). Unraveling the underlying arrhythmia mechanism in persistent atrial fibrillation. *Circ. Arrhythmia Electrophysiol.* 11:e005897. 10.1161/CIRCEP.117.005897 29858382

[B12] Deno D. C. Balachandran R. Morgan D. Ahmad F. Masse S. Nanthakumar K. (2017). Orientation-independent catheter-based characterization of myocardial activation. *IEEE Trans. Biomed. Eng.* 64 1067–1177. 10.1109/TBME.2016.2589158 27411215

[B13] El Haddad M. Houben R. Stroobandt R. Van Heuverswyn F. Tavernier R. Duytschaever M. (2014). Novel algorithmic methods in mapping of atrial and ventricular tachycardia. *Circ. Arrhythmia Electrophysiol.* 7 463–472. 10.1161/CIRCEP.113.000833 24829224

[B14] Gaita F. Caponi D. Scaglione M. Montefusco A. Corleto A. Di Monte F. (2008). Long-term clinical results of 2 different ablation strategies in patients with paroxysmal and persistent atrial fibrillation. *Circ. Arrhythm. Electrophysiol.* 1 269–275. 10.1161/CIRCEP.108.774885 19808418

[B15] Hansen B. J. Zhao J. Li N. Zolotarev A. Zakharkin S. Wang Y. (2018). Human atrial fibrillation drivers resolved with integrated functional and structural imaging to benefit clinical mapping. *JACC Clin. Electrophysiol.* 4 1501–1515. 10.1016/j.jacep.2018.08.024 30573112 PMC6323649

[B16] Herczeg S. Galvin J. Keaney J. J. Keelan E. Byrne R. Howard C. (2020a). The value of voltage histogram analysis derived right atrial scar burden in the prediction of left atrial scar burden. *Cardiol. Res. Pract.* 2020:3981684. 10.1155/2020/3981684 32855820 PMC7442993

[B17] Herczeg S. Walsh K. Keaney J. J. Keelan E. Travers J. Szeplaki G. (2020b). Quantitative assessment of left atrial scar using high-density voltage mapping and a novel automated voltage analysis tool. *J. Interv. Card. Electrophysiol.* 59 5–12. 10.1007/s10840-019-00570-7 31165967

[B18] Hohmann S. Henkenberens C. Zormpas C. Christiansen H. Bauersachs J. Duncker D. (2020). A novel open-source software-based high-precision workflow for target definition in cardiac radioablation. *J. Cardiovasc. Electrophysiol.* 31 2689–2695. 10.1111/jce.14660 32648343

[B19] Iwasaki Y. K. Nishida K. Kato T. Nattel S. (2011). Atrial fibrillation pathophysiology: implications for management. *Circulation* 124 2264–2274. 10.1161/CIRCULATIONAHA.111.019893 22083148

[B20] Jadidi A. S. Lehrmann H. Keyl C. Sorrel J. Markstein V. Minners J. (2016). Ablation of persistent atrial fibrillation targeting low-voltage areas with selective activation characteristics. *Circ. Arrhythmia Electrophysiol.* 9:e002962. 10.1161/CIRCEP.115.002962 26966286

[B21] Jamil-Copley S. Linton N. W. Koa-Wing M. Kojodjojo P. Lim P. B. Malcolme-Lawes L. (2013). Application of ripple mapping with an electroanatomic mapping system for diagnosis of atrial tachycardias. *J. Cardiovasc. Electrophysiol.* 24 1361–1369. 10.1111/jce.12259 24118203

[B22] Kadish A. Johnson D. Choe W. Goldberger J. Horvath G. (2003). Characterization of fibrillatory rhythms by ensemble vector directional analysis. *Am. J. Physiol. Heart Circ. Physiol.* 285 1705–1719. 10.1152/ajpheart.01108.2001 12791595

[B23] Kim Y. H. Chen S. A. Ernst S. Guzman C. E. Han S. Kalarus Z. (2020). 2019 APHRS expert consensus statement on three-dimensional mapping systems for tachycardia developed in collaboration with HRS, EHRA, and LAHRS. *J. Arrhythmia* 36 215–270. 10.1002/joa3.12308 32256872 PMC7132207

[B24] Kistler P. M. Sanders P. Fynn S. P. Stevenson I. H. Spence S. J. Vohra J. K. (2004). Electrophysiologic and electroanatomic changes in the human atrium associated with age. *J. Am. Coll. Cardiol.* 44 109–116. 10.1016/j.jacc.2004.03.044 15234418

[B25] Kojodjojo P. Kanagaratnam P. Markides V. Davies W. Peters N. (2006a). Age-related changes in human left and right atrial conduction. *J. Cardiovasc. Electrophysiol.* 17 120–127. 10.1111/j.1540-8167.2005.00293.x 16533247

[B26] Kojodjojo P. Kanagaratnam P. Segal O. R. Hussain W. Peters N. S. (2006b). The effects of carbenoxolone on human myocardial conduction. a tool to investigate the role of gap junctional uncoupling in human arrhythmogenesis. *J. Am. Coll. Cardiol.* 48 1242–1249. 10.1016/j.jacc.2006.04.093 16979013

[B27] Kojodjojo P. Peters N. S. Davies D. W. Kanagaratnam P. (2007). Characterization of the electroanatomical substrate in human atrial fibrillation: the relationship between changes in atrial volume, refractoriness, wavefront propagation velocities, and AF burden. *J. Cardiovasc. Electrophysiol.* 18 269–275. 10.1111/j.1540-8167.2007.00723.x 17318996

[B28] Lau D. H. Linz D. Sanders P. (2019). New findings in atrial fibrillation mechanisms. *Card. Electrophysiol. Clin.* 11 563–571. 10.1016/j.ccep.2019.08.007 31706465

[B29] Linton N. W. Koa-Wing M. Francis D. P. Kojodjojo P. Lim P. B. Salukhe T. V. (2009). Cardiac ripple mapping: a novel three-dimensional visualization method for use with electroanatomic mapping of cardiac arrhythmias. *Heart Rhythm* 6 1754–1762. 10.1016/j.hrthm.2009.08.038 19959125

[B30] Lucivero F. (2020). Big data, big waste? a reflection on the environmental sustainability of big data initiatives. *Sci. Eng. Ethics* 26 1009–1030. 10.1007/s11948-019-00171-7 31893331 PMC7089892

[B31] Masè M. Ravelli F. (2010). “Automatic reconstruction of activation and velocity maps from electro-anatomic data by radial basis functions,” in *In Procceings of the 2010 Annual International Conference IEEE Engineering Medical Biology Social*, (Piscataway, NJ: IEEE). 10.1109/IEMBS.2010.5626616 21096180

[B32] Massé S. Magtibay K. Jackson N. Asta J. Kusha M. Zhang B. (2016). Resolving myocardial activation with novel omnipolar electrograms. *Circ. Arrhythmia Electrophysiol.* 9:e004107. 10.1161/CIRCEP.116.004107 27406608 PMC4956680

[B33] Mourad A. Nash M. (2007). Method for quantifiying conduction velocity during ventricular fibrillation. *Phys. Rev. E Stat. Nonlin. Soft Matter Phys.* 75:011914. 10.1103/PhysRevE.75.011914 17358191

[B34] Nalliah C. J. Wong G. R. Lee G. Voskoboinik A. Kee K. Goldin J. (2021). Sleep apnoea has a dose-dependent effect on atrial remodelling in paroxysmal but not persistent atrial fibrillation: a high-density mapping study. *Europace* euaa275. 10.1093/europace/euaa275 33447844

[B35] O’Neill L. Karim R. Mukherjee R. K. Whitaker J. Sim I. Harrison J. (2019). Pulmonary vein encirclement using an ablation index-guided point-by-point workflow: cardiovascular magnetic resonance assessment of left atrial scar formation. *Europace* 21 1817–1823. 10.1093/europace/euz226 31793653 PMC6887923

[B36] Pak H.-N. Oh Y. S. Lim H. E. Kim Y.-H. Hwang C. (2011). Comparison of voltage map-guided left atrial anterior wall ablation versus left lateral mitral isthmus ablation in patients with persistent atrial fibrillation. *Heart Rhythm* 8 199–206. 10.1016/j.hrthm.2010.10.015 20950713

[B37] Pappone C. Mecarocci V. Manguso F. Ciconte G. Vicedomini G. Sturla F. (2020). New electromechanical substrate abnormalities in high-risk patients with Brugada syndrome. *Heart Rhythm* 17 637–645. 10.1016/j.hrthm.2019.11.019 31756528

[B38] Pokorney S. D. Friedman D. J. Calkins H. Callans D. J. Daoud E. G. Della-Bella P. (2016). Catheter ablation of ventricular tachycardia: lessons learned from past clinical trials and implications for future clinical trials. *Heart Rhythm* 13 1748–1754. 10.1016/j.hrthm.2016.04.001 27050910 PMC5070490

[B39] Ravelli F. Masè M. Del Greco M. Marini M. Disertori M. (2011). Acute atrial dilatation slows conduction and increases AF vulnerability in the human atrium. *J. Cardiovasc. Electrophysiol.* 22 394–401. 10.1111/j.1540-8167.2010.01939.x 21044210

[B40] Ríos-Muñoz G. R. Arenal Á Artés-Rodríguez A. (2018). Real-time rotational activity detection in atrial fibrillation. *Front. Physiol.* 9:208. 10.3389/fphys.2018.01260 29593566 PMC5859379

[B41] Roney C. H. Cantwell C. D. Qureshi N. A. Ali R. L. Chang E. T. Y. Lim P. B. (2014). “An automated algorithm for determining conduction velocity, wavefront direction and origin of focal cardiac arrhythmias using a multipolar catheter,” in *In Procceings of the 2014 36th Annual International Conference IEEE Engineering Medical Biology Social*, (Piscataway, NJ: IEEE). 10.1109/EMBC.2014.6943906 25570274

[B42] Roney C. H. Whitaker J. Sim I. O’Neill L. Mukherjee R. K. Razeghi O. (2019). A technique for measuring anisotropy in atrial conduction to estimate conduction velocity and atrial fibre direction. *Comput. Biol. Med.* 104 278–290. 10.1016/j.compbiomed.2018.10.019 30415767 PMC6506689

[B43] Sawa A. Shimizu A. Ueyama T. Yoshiga Y. Suzuki S. Sugi N. (2008). Activation patterns and conduction velocity in posterolateral right atrium during typical atrial flutter using an electroanatomic mapping system. *Circ. J.* 72 384–391. 10.1253/circj.72.384 18296833

[B44] Taghji P. El Haddad M. Phlips T. Wolf M. Knecht S. Vandekerckhove Y. (2018). Evaluation of a strategy aiming to enclose the pulmonary veins with contiguous and optimized radiofrequency lesions in paroxysmal atrial fibrillation. *JACC Clin. Electrophysiol.* 4 99–108. 10.1016/j.jacep.2017.06.023 29600792

[B45] Vraka A. Hornero F. Bertomeu-González V. Osca J. Alcaraz R. Rieta J. J. (2020). Short-time estimation of fractionation in atrial fibrillation with coarse-grained correlation dimension for mapping the atrial substrate. *Entropy* 22:232. 10.3390/e22020232 33286006 PMC7516661

[B46] Weber F. M. Luik A. Schilling C. Seemann G. Krueger M. W. Lorenz C. (2011). Conduction velocity restitution of the human atriuman-An efficient measurement protocol for clinical electrophysiological studies. *IEEE Trans. Biomed. Eng.* 58 2648–2655. 10.1109/TBME.2011.2160453 21708491

[B47] Whitaker J. Fish J. Harrison J. L. Chubb H. Williams S. E. Fastl T. (2018). Lesion index-guided ablation facilitates continuous, transmural, and durable lesions in a porcine recovery model. *Circ. Arrhythmia Electrophysiol.* 11:e005892. 10.1161/CIRCEP.117.005892 29654131

[B48] Williams S. E. Linton N. W. F. (2021). *Openep/Openep-Core: First OpenEP Release (Version v1.0.0).*

[B49] Williams S. E. Harrison J. L. Chubb H. Whitaker J. Kiedrowicz R. Rinaldi C. A. C. A. (2018). Local activation time sampling density for atrial tachycardia contact mapping: how much is enough. *Europace* 20 e11–e20. 10.1093/europace/eux037 28379525 PMC5834039

[B50] Williams S. E. Linton N. W. Harrison J. L. Chubb H. Whitaker J. Gill J. S. (2017). Intra-atrial conduction delay revealed by multisite incremental atrial pacing is an independent marker of remodeling in human atrial fibrillation. *JACC Clin. Electrophysiol.* 3 1006–1017. 10.1016/j.jacep.2017.02.012 28966986 PMC5612260

[B51] Williams S. E. O’Neill L. Roney C. H. C. H. Julia J. Metzner A. Reißmann B. (2019). Left atrial effective conducting size predicts atrial fibrillation vulnerability in persistent but not paroxysmal atrial fibrillation. *J. Cardiovasc. Electrophysiol.* 30 1416–1427. 10.1111/jce.13990 31111557 PMC6746623

