# Supplementary material for: OpenEP: A Cross-Platform Electroanatomic Mapping Data Format and Analysis Platform for Electrophysiology Research
Source: Front Physiol. 2021 Feb 26;12:646023. doi: 10.3389/fphys.2021.646023 (PMC7952326; doi:10.3389/fphys.2021.646023)
Supplement: Supplementary file 1 [file Data_Sheet_1.PDF]

Road map for future development (to be published on website)

Infrastructure	Data parsing	Data analysis
Octave-based implementation<br>Graphical interface<br>Conversation to object-orientated format	Rhythmia data parsing	Full 12-lead ECG data<br>Fractionation indices<br>Segmental atrial analysis<br>Segmental ventricular analysis<br>Late potential mapping<br>Local activation histogram analysis
