# Supplementary material for: OpenEP: A Cross-Platform Electroanatomic Mapping Data Format and Analysis Platform for Electrophysiology Research
Source: Front Physiol. 2021 Feb 26;12:646023. doi: 10.3389/fphys.2021.646023 (PMC7952326; doi:10.3389/fphys.2021.646023)
Supplement: Supplementary file 2 [file Data_Sheet_2.PDF]

Supplementary Material

Full Literature Search Result

Search: (electroanatomic mapping) AND ((atrial fibrillation) OR (ventricular tachycardia)) Filters: 1 year. Search performed on 9th November 2020.

1: Guandalini GS, Liang JJ, Marchlinski FE. Ventricular Tachycardia Ablation: Past, Present, and Future Perspectives. JACC Clin Electrophysiol. 2019 Dec;5(12):1363-1383. doi: 10.1016/j.jacep.2019.09.015. PMID: 31857035.

2: Kautzner J, Peichl P. Mapping and Ablation of Unmappable Ventricular Tachycardia, Ventricular Tachycardia Storm, and Those in Acute Myocardial Infarction. Card Electrophysiol Clin. 2019 Dec;11(4):675-688. doi: 10.1016/j.ccep.2019.08.006. PMID: 31706474.

3: Romero J, Natale A, Lakkireddy D, Cerna L, Diaz JC, Alviz I, Cerrud-Rodriguez RC, Grupposo V, Rios SA, Chernobelsky E, Elsayed MG, Garcia M, Di Biase L. Mapping and localization of the left phrenic nerve during left atrial appendage electrical isolation to avoid inadvertent injury in patients undergoing catheter ablation of atrial fibrillation. Heart Rhythm. 2020 Apr;17(4):527-534. doi: 10.1016/j.hrthm.2019.10.020. Epub 2019 Oct 18. PMID: 31634618.

4: Jan M, Žižek D, Kuhelj D, Lakič N, Prolič Kalinšek T, Štublar J, Klemen L, Pernat A, Antolič B. Combined use of electro-anatomic mapping system and intracardiac echocardiography to achieve zero-fluoroscopy catheter ablation for treatment of paroxysmal atrial fibrillation: a single centre experience. Int J Cardiovasc Imaging. 2020 Mar;36(3):415-422. doi: 10.1007/s10554-019-01727-1. Epub 2019 Nov 11. PMID: 31712932.

5: Tung R. Substrate Mapping in Ventricular Arrhythmias. Card Electrophysiol Clin. 2019 Dec;11(4):657-663. doi: 10.1016/j.ccep.2019.08.009. PMID: 31706472.

6: Haanschoten DM, Adiyaman A, Smit JJJ, Delnoy PPHM, Ramdat Misier AR, Porta F, Storm van Leeuwen RPH, Elvan A. Hybrid Ventricular Tachycardia Ablation after Failed Percutaneous Endocardial and Epicardial Ablation. Cardiology. 2020;145(2):88-94. doi: 10.1159/000503251. Epub 2019 Nov 8. PMID: 31707389.

7: Cronin EM, Bogun FM, Maury P, Peichl P, Chen M, Namboodiri N, Aguinaga L, Leite LR, Al-Khatib SM, Anter E, Berruezo A, Callans DJ, Chung MK, Cuculich P, d'Avila A, Deal BJ, Della Bella P, Deneke T, Dickfeld TM, Hadid C, Haqqani HM, Kay GN, Latchamsetty R, Marchlinski F, Miller JM, Nogami A, Patel AR, Pathak RK, Saenz Morales LC, Santangeli P, Sapp JL Jr, Sarkozy A, Soejima K, Stevenson WG, Tedrow UB, Tzou WS, Varma N, Zeppenfeld K. 2019 HRS/EHRA/APHRS/LAHRS expert consensus statement on catheter ablation of ventricular arrhythmias. Heart Rhythm. 2020 Jan;17(1):e2-e154. doi: 10.1016/j.hrthm.2019.03.002. Epub 2019 May 10. PMID: 31085023.

8: Pappone C, Mecarocci V, Manguso F, Ciconte G, Vicedomini G, Sturla F, Votta E, Mazza B, Pozzi P, Borrelli V, Anastasia L, Micaglio E, Locati E, Monasky MM, Lombardi M, Calovic Z, Santinelli V. New electromechanical substrate abnormalities in high-risk patients with Brugada syndrome. Heart Rhythm. 2020 Apr;17(4):637-645. doi: 10.1016/j.hrthm.2019.11.019. Epub 2019 Nov 19. PMID: 31756528.

9: Neuzner J, Dietze T, Paliege R, Gradaus R. Effectiveness of a percutaneous left ventricular assist device in preventing acute hemodynamic decompensation during catheter ablation of ventricular tachycardia in advanced heart failure patients: A retrospective single-center analysis. J Cardiovasc Electrophysiol. 2019 Dec;30(12):2864-2868. doi: 10.1111/jce.14199. Epub 2019 Oct 2. PMID: 31549434.

10: Jiang R, Beaser AD, Aziz Z, Upadhyay GA, Nayak HM, Tung R. High-Density Grid Catheter for Detailed Mapping of Sinus Rhythm and Scar-Related Ventricular Tachycardia: Comparison With a Linear Duodecapolar Catheter. JACC Clin Electrophysiol. 2020 Mar;6(3):311-323. doi: 10.1016/j.jacep.2019.11.007. Epub 2020 Jan 29. PMID: 32192682.

11: Cronin EM, Bogun FM, Maury P, Peichl P, Chen M, Namboodiri N, Aguinaga L, Leite LR, Al-Khatib SM, Anter E, Berruezo A, Callans DJ, Chung MK, Cuculich P, d'Avila A, Deal BJ, Della Bella P, Deneke T, Dickfeld TM, Hadid C, Haqqani HM, Kay GN, Latchamsetty R, Marchlinski F, Miller JM, Nogami A, Patel AR, Pathak RK, Saenz Morales LC, Santangeli P, Sapp JL Jr, Sarkozy A, Soejima K, Stevenson WG, Tedrow UB, Tzou WS, Varma N, Zeppenfeld K. 2019 HRS/EHRA/APHRS/LAHRS expert consensus statement on catheter ablation of ventricular arrhythmias: Executive summary. Heart Rhythm. 2020 Jan;17(1):e155-e205. doi: 10.1016/j.hrthm.2019.03.014. Epub 2019 May 10. PMID: 31102616.

12: Berte B, Cochet H, Dang L, Mahida S, Moccetti F, Hilfiker G, Bondietti J, Ruschitzka F, Jaïs P, Scharf C, Kobza R. Image-guided ablation of scar-related ventricular tachycardia: towards a shorter and more predictable procedure. J Interv Card Electrophysiol. 2019 Dec 19. doi: 10.1007/s10840-019-00686-w. Epub ahead of print. PMID: 31858334.

13: Bourier F, Vlachos K, Lam A, Martin CA, Takigawa M, Kitamura T, Massoullié G, Cheniti G, Frontera A, Duchateau J, Pambrun T, Klotz N, Derval N, Denis A, Hocini M, Haïssaguerre M, Cochet H, Jaïs P, Sacher F. Three-dimensional image integration guidance for cryoballoon pulmonary vein isolation procedures. J Cardiovasc Electrophysiol. 2019 Dec;30(12):2790-2796. doi: 10.1111/jce.14249. Epub 2019 Oct 31. PMID: 31646698.

14: Peretto G, Sala S, Rizzo S, Palmisano A, Esposito A, De Cobelli F, Campochiaro C, De Luca G, Foppoli L, Dagna L, Thiene G, Basso C, Della Bella P. Ventricular Arrhythmias in Myocarditis: Characterization and Relationships With Myocardial Inflammation. J Am Coll Cardiol. 2020 Mar 10;75(9):1046-1057. doi: 10.1016/j.jacc.2020.01.036. PMID: 32138965.

15: Aksu T, Guler TE. Electroanatomical mapping-guided ablation during atrial fibrillation: a novel usage of fractionation mapping in a case with sinus bradycardia and paroxysmal atrial fibrillation. J Interv Card Electrophysiol. 2020 Mar;57(2):331-332. doi: 10.1007/s10840-019-00633-9. Epub 2019 Nov 9. PMID: 31707532.

16: Yoshida K, Baba M, Hasebe H, Shinoda Y, Harunari T, Ebine M, Uehara Y, Watabe H, Takeyasu N, Horigome H, Nogami A, Ieda M. Structural relation between the superior vena cava and pulmonary veins in patients with atrial fibrillation. Heart Vessels. 2019 Dec;34(12):2052-2058. doi: 10.1007/s00380-019-01431-z. Epub 2019 May 22. PMID: 31114962.

17: Krug D, Blanck O, Demming T, Dottermusch M, Koch K, Hirt M, Kotzott L, Zaman A, Eidinger L, Siebert FA, Dunst J, Bonnemeier H. Stereotactic body radiotherapy for ventricular tachycardia (cardiac radiosurgery) : First-in-patient treatment in Germany. Strahlenther Onkol. 2020 Jan;196(1):23-30. doi: 10.1007/s00066-019-01530-w. Epub 2019 Oct 31. PMID: 31673718.

18: Reddy VY, Schilling R, Grimaldi M, Horton R, Natale A, Riva S, Tondo C, Kuck KH, Neuzil P, McInnis K, Bishara M, Zhang B, Govari A, Abdelaal A, Mansour M. Pulmonary Vein Isolation With a Novel Multielectrode Radiofrequency Balloon Catheter That Allows Directionally Tailored Energy Delivery: Short-Term Outcomes From a Multicenter First-in-Human Study (RADIANCE). Circ Arrhythm Electrophysiol. 2019 Dec;12(12):e007541. doi: 10.1161/CIRCEP.119.007541. Epub 2019 Dec 12. PMID: 31826648.

19: Canpolat U, Mohanty S, Trivedi C, Chen Q, Ayhan H, Gianni C, Della Rocca DG, MacDonald B, Burkhardt JD, Bassiouny M, Gallinghouse GJ, Al-Ahmad A, Horton R, Di Biase L, Natale A. Association of fragmented QRS with left atrial scarring in patients with persistent atrial fibrillation undergoing

radiofrequency catheter ablation. Heart Rhythm. 2020 Feb;17(2):203-210. doi: 10.1016/j.hrthm.2019.09.010. Epub 2019 Sep 10. PMID: 31518722.

20: Briceño DF, Liang JJ, Shirai Y, Markman TM, Chahal A, Tschabrunn C, Zado E, Hyman MC, Kumareswaran R, Arkles JS, Santangeli P, Schaller RD, Supple GE, Frankel DS, Deo R, Riley MP, Nazarian S, Lin D, Epstein AE, Garcia FC, Dixit S, Callans DJ, Marchlinski FE. Characterization of Structural Changes in Arrhythmogenic Right Ventricular Cardiomyopathy With Recurrent Ventricular Tachycardia After Ablation: Insights From Repeat Electroanatomic Voltage Mapping. Circ Arrhythm Electrophysiol. 2020 Jan;13(1):e007611. doi: 10.1161/CIRCEP.119.007611. Epub 2020 Jan 10. PMID: 31922914.

21: Cronin EM, Bogun FM, Maury P, Peichl P, Chen M, Namboodiri N, Aguinaga L, Leite LR, Al-Khatib SM, Anter E, Berruezo A, Callans DJ, Chung MK, Cuculich P, d'Avila A, Deal BJ, Bella PD, Deneke T, Dickfeld TM, Hadid C, Haqqani HM, Kay GN, Latchamsetty R, Marchlinski F, Miller JM, Nogami A, Patel AR, Pathak RK, Saenz Morales LC, Santangeli P, Sapp JL Jr, Sarkozy A, Soejima K, Stevenson WG, Tedrow UB, Tzou WS, Varma N, Zeppenfeld K. 2019 HRS/EHRA/APHRS/LAHRS expert consensus statement on catheter ablation of ventricular arrhythmias. J Interv Card Electrophysiol. 2020 Oct;59(1):145-298. doi: 10.1007/s10840-019-00663-3. PMID: 31984466; PMCID: PMC7223859.

22: Tofig BJ, Lukac P, Nielsen JM, Hansen ESS, Tougaard RS, Jensen HK, Nielsen JC, Kristiansen SB. Radiofrequency ablation lesions in low-, intermediate-, and normal-voltage myocardium: an in vivo study in a porcine heart model. Europace. 2019 Dec 1;21(12):1919-1927. doi: 10.1093/europace/euz247. PMID: 31545375.

23: Wang XH, Li Z, Zang MH, Yao TB, Mao JL, Pu J. Circulating primary bile acid is correlated with structural remodeling in atrial fibrillation. J Interv Card Electrophysiol. 2020 Apr;57(3):371-377. doi: 10.1007/s10840-019-00540-z. Epub 2019 Mar 26. PMID: 30915593.

24: Almeida T, Soriano D, Mase M, Ravelli F, Bezerra A, Li X, Chu G, Salinet J, Stafford P, Andre Ng G, Schlindwein F, Yoneyama T. Unsupervised Classification of Atrial Electrograms for Electroanatomic Mapping of Human Persistent Atrial Fibrillation. IEEE Trans Biomed Eng. 2020 Sep 3;PP. doi: 10.1109/TBME.2020.3021480. Epub ahead of print. PMID: 32881680.

25: Elbatran AI, Leung LWM, Gallagher MM. Left atrial appendage isolation during ablation in the interatrial septum: Rapid recognition by continuous monitoring of appendage electrograms. Pacing Clin Electrophysiol. 2020 Jan;43(1):149-152. doi: 10.1111/pace.13861. Epub 2019 Dec 23. PMID: 31849083.

26: Choudry S, Mansour M, Sundaram S, Nguyen DT, Dukkipati SR, Whang W, Kessman P, Reddy VY. RADAR: A Multicenter Food and Drug Administration Investigational Device Exemption Clinical Trial of Persistent Atrial Fibrillation. Circ Arrhythm Electrophysiol. 2020 Jan;13(1):e007825. doi: 10.1161/CIRCEP.119.007825. Epub 2020 Jan 16. PMID: 31944826; PMCID: PMC6970579.

27: Jiang CX, Long DY, Du X, Sang CH, Yao Y, Li MM, Tang RB, Li SN, Wen SN, Bai R, Dong JZ, Ma CS. Atrial tachycardia eliminated at the ventricular side in patients with congenitally corrected transposition of the great arteries: Electrophysiological findings and anatomical concerns. Heart Rhythm. 2020 Aug;17(8):1337-1345. doi: 10.1016/j.hrthm.2020.03.010. Epub 2020 Mar 20. PMID: 32201269.

28: Compagnucci P, Volpato G, Falanga U, Cipolletta L, Conti M, Grifoni G, Verticelli L, Schicchi N, Giovagnoni A, Casella M, Guerra F, Dello Russo A. Recent advances in three-dimensional electroanatomical mapping guidance for the ablation of complex atrial and ventricular arrhythmias. J Interv Card Electrophysiol. 2020 May 26. doi: 10.1007/s10840-020-00781-3. Epub ahead of print. PMID: 32451799.

29: Herczeg S, Walsh K, Keaney JJ, Keelan E, Travers J, Szeplaki G, Galvin J. Quantitative assessment of left atrial scar using high-density voltage mapping and a novel automated voltage analysis tool. J Interv Card Electrophysiol. 2020 Oct;59(1):5-12. doi: 10.1007/s10840-019-00570-7. Epub 2019 Jun 5. PMID: 31165967.

30: Kochar A, Ahmed T, Donnellan E, Wazni O, Tchou P, Chung R. Operator learning curve and clinical outcomes of zero fluoroscopy catheter ablation of atrial fibrillation, supraventricular tachycardia, and ventricular arrhythmias. J Interv Card Electrophysiol. 2020 Jun 12. doi: 10.1007/s10840-020-00798-8. Epub ahead of print. PMID: 32529314.

31: Takagi T, Nakamura K, Hashimoto H, Asami M, Ishii R, Enomoto Y, Moroi M, Noro M, Sugi K, Nakamura M. The impact of sleep apnea on right atrial structural remodeling with atrial fibrillation. J Cardiol. 2020 Jun;75(6):665-672. doi: 10.1016/j.jjcc.2019.12.012. Epub 2020 Jan 20. PMID: 31973979.

32: Brett CL, Cook JA, Aboud AA, Karim R, Shinohara ET, Stevenson WG. Novel Workflow for Conversion of Catheter-Based Electroanatomic Mapping to DICOM Imaging for Noninvasive Radioablation of Ventricular Tachycardia. Pract Radiat Oncol. 2020 May 13:S1879-8500(20)30103-X. doi: 10.1016/j.prro.2020.04.006. Epub ahead of print. PMID: 32416269.

33: Lin R, Wu S, Zhu D, Qin M, Liu X. Osteopontin induces atrial fibrosis by activating Akt/GSK-3β/β-catenin pathway and suppressing autophagy. Life Sci. 2020 Mar 15;245:117328. doi: 10.1016/j.lfs.2020.117328. Epub 2020 Jan 15. PMID: 31954162.

34: Bennett R, Campbell T, Eslick A, Pudipeddi A, Hing A, Yeates L, Hanna B, Kumar S. Ventricular Tachycardia in a Patient With Dilated Cardiomyopathy Caused by a Novel Mutation of Lamin A/C Gene: Insights From Features on Electroanatomic Mapping, Catheter Ablation and Tissue Pathology. Heart Lung Circ. 2020 Oct 5:S1443-9506(20)30478-9. doi: 10.1016/j.hlc.2020.08.024. Epub ahead of print. PMID: 33032896.

35: Huang D, Sun Y, Lin K, Li F, Pan JW, Li JB, Wei M. Repetitive ventricular tachycardia triggered by a giant benign cardiac lipomyoma. J Electrocardiol. 2020 Jan-Feb;58:29-32. doi: 10.1016/j.jelectrocard.2019.10.006. Epub 2019 Oct 20. PMID: 31678719.

36: Sakamoto SI, Hiromoto A, Murata H, Suzuki K, Kurita J, Kawase Y, Sasaki T, Miyagi Y, Ishii Y, Morota T, Shimizu W, Nitta T. Surgical procedure for targeting arrhythmogenic substrates in the treatment of ventricular tachycardia associated with cardiac tumors. Heart Rhythm. 2020 Feb;17(2):238-242. doi: 10.1016/j.hrthm.2019.08.025. Epub 2019 Aug 30. PMID: 31476412.

37: Kitamura T, Takigawa M, Derval N, Denis A, Martin R, Vlachos K, Nakatani Y, Frontera A, Cheniti G, Martin CA, Bourier F, Lam A, Duchateau J, Pambrun T, Sacher F, Cochet H, Hocini M, Haïssaguerre M, Jaïs P. Atrial tachycardia circuits include low voltage area from index atrial fibrillation ablation relationship between RF ablation lesion and AT. J Cardiovasc Electrophysiol. 2020 Jul;31(7):1640-1648. doi: 10.1111/jce.14576. Epub 2020 Jun 1. PMID: 32437007.

38: Rivera S, Vecchio N, Ricapito P, Ayala-Paredes F. Non-fluoroscopic catheter ablation of arrhythmias with origin at the summit of the left ventricle. J Interv Card Electrophysiol. 2019 Dec;56(3):279-290. doi: 10.1007/s10840-019-00522-1. Epub 2019 Feb 7. PMID: 30729374.

39: Romero J, Natale A, Cerna L, Lakkireddy D, Diaz JC, Alviz I, Grupposo V, Rios SA, Chernobelsky E, Lopez Cabanillas N, Garcia M, Di Biase L. Imaging Integration to Localize and Protect the Left Coronary Artery in Patients Undergoing LAAEI. JACC Clin Electrophysiol. 2020 Feb;6(2):157-167. doi: 10.1016/j.jacep.2019.09.011. Epub 2019 Nov 27. PMID: 32081217.

40: La Greca C, Cirasa A, Di Modica D, Sorgato A, Simoncelli U, Pecora D. Advantages of the integration of ICE and 3D electroanatomical mapping and ultrasound-guided femoral venipuncture in catheter ablation of atrial fibrillation. J Interv Card Electrophysiol. 2020 Aug 18. doi: 10.1007/s10840-020-00835-6. Epub ahead of print. PMID: 32809149.

41: Cronin EM, Bogun FM, Maury P, Peichl P, Chen M, Namboodiri N, Aguinaga L, Leite LR, Al-Khatib SM, Anter E, Berruezo A, Callans DJ, Chung MK, Cuculich P, d'Avila A, Deal BJ, Della Bella P, Deneke T, Dickfeld TM, Hadid C, Haqqani HM, Kay GN, Latchamsetty R, Marchlinski F, Miller JM, Nogami A, Patel AR, Pathak RK, Saenz Morales LC, Santangeli P, Sapp JL, Sarkozy A, Soejima K, Stevenson WG, Tedrow UB, Tzou WS, Varma N, Zeppenfeld K. 2019 HRS/EHRA/APHRS/LAHRS expert consensus statement on catheter ablation of ventricular arrhythmias: executive summary. Europace. 2020 Mar 1;22(3):450-495. doi: 10.1093/europace/euz332. PMID: 31995197.

42: Cronin EM, Bogun FM, Maury P, Peichl P, Chen M, Namboodiri N, Aguinaga L, Leite LR, Al-Khatib SM, Anter E, Berruezo A, Callans DJ, Chung MK, Cuculich P, d'Avila A, Deal BJ, Bella PD, Deneke T, Dickfeld TM, Hadid C, Haqqani HM, Kay GN, Latchamsetty R, Marchlinski F, Miller JM, Nogami A, Patel AR, Pathak RK, Saenz Morales LC, Santangeli P, Sapp JL Jr, Sarkozy A, Soejima K, Stevenson WG, Tedrow UB, Tzou WS, Varma N, Zeppenfeld K. 2019 HRS/EHRA/APHRS/LAHRS expert consensus statement on catheter ablation of ventricular arrhythmias: Executive summary. J Arrhythm. 2020 Jan 3;36(1):1-58. doi: 10.1002/joa3.12264. PMID: 32071620; PMCID: PMC7011820.

43: Rivner H, Mitrani RD, Goldberger JJ. Atrial Myopathy Underlying Atrial Fibrillation. Arrhythm Electrophysiol Rev. 2020 Aug;9(2):61-70. doi: 10.15420/aer.2020.13. PMID: 32983526; PMCID: PMC7491052.

44: Aronis KN, Ali RL, Prakosa A, Ashikaga H, Berger RD, Hakim JB, Liang J, Tandri H, Teng F, Chrispin J, Trayanova NA. Accurate Conduction Velocity Maps and Their Association With Scar Distribution on Magnetic Resonance Imaging in Patients With Postinfarction Ventricular Tachycardias. Circ Arrhythm Electrophysiol. 2020 Apr;13(4):e007792. doi: 10.1161/CIRCEP.119.007792. Epub 2020 Mar 19. PMID: 32191131; PMCID: PMC7196439.

45: Campbell T, Trivic I, Bennett RG, Anderson RD, Turnbull S, Pham T, Nalliah C, Kizana E, Watts T, Lee G, Kumar S. Catheter ablation of ventricular arrhythmia guided by a high-density grid catheter. J Cardiovasc Electrophysiol. 2020 Feb;31(2):474-484. doi: 10.1111/jce.14351. Epub 2020 Jan 20. PMID: 31930658.

46: Parwani AS, Hohendanner F, Boldt LH. Katheterablation bei ventrikulärer Tachyarrhythmie : Klinische Ergebnisse [Catheter ablation of ventricular tachycardia : Clinical outcome]. Herzschrittmacherther Elektrophysiol. 2019 Dec;30(4):349-355. German. doi: 10.1007/s00399-019-00653-y. Epub 2019 Nov 11. PMID: 31713027.

47: Zhou S, Sapp JL, Horáček BM, Warren JW, MacInnis PJ, Davis J, Elsokkari I, Choudhury R, Parkash R, Gray C, Gardner M, MacIntyre CJ, AbdelWahab A. Automated intraprocedural localization of origin of ventricular activation using patient- specific computed tomographic imaging. Heart Rhythm. 2020 Apr;17(4):567-575. doi: 10.1016/j.hrthm.2019.10.025. Epub 2019 Oct 25. PMID: 31669770.

48: Kella DK, Sheldon SH, Noheria A, Padmanabhan D, Munger T, Asirvatham SJ, Kapa S. Defining the substrate for ventricular tachycardia ablation: The impact of rhythm at the time of mapping. Indian Pacing Electrophysiol J. 2020 Jul- Aug;20(4):147-153. doi: 10.1016/j.ipej.2020.03.005. Epub 2020 Mar 7. PMID: 32156640; PMCID: PMC7371953.

49: Boersma L, Rienstra M, de Groot JR. Therapeutic options for patients with advanced atrial fibrillation: from lifestyle and medication to catheter and surgical ablation. Neth Heart J. 2020 Aug;28(Suppl 1):13-18. doi: 10.1007/s12471-020-01447-5. PMID: 32780326; PMCID: PMC7419415.

50: Roney CH, Beach ML, Mehta AM, Sim I, Corrado C, Bendikas R, Solis-Lemus JA, Razeghi O, Whitaker J, O'Neill L, Plank G, Vigmond E, Williams SE, O'Neill MD, Niederer SA. *In silico* Comparison of Left Atrial Ablation Techniques That Target the Anatomical, Structural, and Electrical Substrates of Atrial Fibrillation. Front Physiol. 2020 Sep 16;11:1145. doi: 10.3389/fphys.2020.572874. PMID: 33041850; PMCID: PMC7526475.

51: Eichenlaub M, Weber R, Minners J, Allgeier HJ, Jadidi A, Müller-Edenborn B, Neumann FJ, Arentz T, Lehrmann H. 3D mapping for the identification of the fossa ovalis in left atrial ablation procedures: a pilot study of a first step towards an electroanatomic-guided transseptal puncture. Europace. 2020 May 1;22(5):732-738. doi: 10.1093/europace/euaa034. PMID: 32142124.

52: Pang L, Chen SW, Zhou GQ, Wei Y, Chen C, Huang SA, Liu SW. A Practical Method for Ablation Catheter Reintroduction into the Left Atrium via Prior Transseptal Puncture, without Radiation. Heart Surg Forum. 2019 Dec 3;22(6):E470-E475. doi: 10.1532/hsf.2621. PMID: 31895032.

53: Mayinger M, Kovacs B, Tanadini-Lang S, Ehrbar S, Wilke L, Chamberlain M, Moreira A, Weitkamp N, Brunckhorst C, Duru F, Steffel J, Breitenstein A, Alkadhi H, Garcia Schueler HI, Manka R, Ruschitzka F, Guckenberger M, Saguner AM, Andratschke N. First magnetic resonance imaging-guided cardiac radioablation of sustained ventricular tachycardia. Radiother Oncol. 2020 Feb 14:S0167-8140(20)30020-7. doi: 10.1016/j.radonc.2020.01.008. Epub ahead of print. PMID: 32067819.

54: Grubb CS, Melki L, Wang DY, Peacock J, Dizon J, Iyer V, Sorbera C, Biviano A, Rubin DA, Morrow JP, Saluja D, Tieu A, Nauleau P, Weber R, Chaudhary S, Khurram I, Waase M, Garan H, Konofagou EE, Wan EY. Noninvasive localization of cardiac arrhythmias using electromechanical wave imaging. Sci Transl Med. 2020 Mar 25;12(536):eaax6111. doi: 10.1126/scitranslmed.aax6111. PMID: 32213631; PMCID: PMC7234276.

55: Graham AJ, Orini M, Zacur E, Dhillon G, Daw H, Srinivasan NT, Martin C, Lane J, Mansell JS, Cambridge A, Garcia J, Pugliese F, Segal O, Ahsan S, Lowe M, Finlay M, Earley MJ, Chow A, Sporton S, Dhinoja M, Hunter RJ, Schilling RJ, Lambiase PD. Evaluation of ECG Imaging to Map Hemodynamically Stable and Unstable Ventricular Arrhythmias. Circ Arrhythm Electrophysiol. 2020 Feb;13(2):e007377. doi: 10.1161/CIRCEP.119.007377. Epub 2020 Jan 14. PMID: 31934784.

56: Koruth JS, Kuroki K, Iwasawa J, Viswanathan R, Brose R, Buck ED, Donskoy E, Dukkipati SR, Reddy VY. Endocardial ventricular pulsed field ablation: a proof- of-concept preclinical evaluation. Europace. 2020 Mar 1;22(3):434-439. doi: 10.1093/europace/euz341. PMID: 31876913; PMCID: PMC7058968.

57: Sun Y, Yu X, Xiao X, Yin X, Gao L, Zhang R, Dai S, Wang N, Zhang D, Dong Y, Yang Y, Xia Y. High efficiency and workflow of His bundle pacing and atrioventricular node ablation guided by three-dimensional mapping system. Pacing Clin Electrophysiol. 2020 Oct;43(10):1165-1172. doi: 10.1111/pace.14061. Epub 2020 Sep 22. PMID: 32896924.

58: Hadjis A, Frontera A, Limite LR, Bisceglia C, Bognoni L, Foppoli L, Lipartiti F, Paglino G, Radinovic A, Tsitsinakis G, Calore F, Della Bella P. Complete Electroanatomic Imaging of the Diastolic Pathway Is Associated With Improved Freedom From Ventricular Tachycardia Recurrence. Circ Arrhythm Electrophysiol. 2020 Sep;13(9):e008651. doi: 10.1161/CIRCEP.120.008651. Epub 2020 Jul 28. PMID: 32755381; PMCID: PMC7495983.

59: Paço P, Tura B, Santos M, Amparo P, De Lorenzo A. Budget Impact of Cryoablation Versus Radiofrequency Ablation of Atrial Fibrillation in the Brazilian Public Healthcare System. Value Health Reg Issues. 2019 Dec;20:149-153. doi: 10.1016/j.vhri.2019.05.004. Epub 2019 Aug 21. PMID: 31445328.

60: Patil KD, Chrispin J. Ventricular Tachycardia Ablation in Patients with Left Ventricular Assist Devices. J Innov Card Rhythm Manag. 2019 Nov 15;10(11):3913-3918. doi: 10.19102/icrm.2019.101101. PMID: 32477712; PMCID: PMC7252754.

61: Romero J, Patel K, Briceno D, Alviz I, Tarantino N, Della Rocca DG, Natale V, Zhang XD, Di Biase L. Fluoroless Atrial Fibrillation Catheter Ablation: Technique and Clinical Outcomes. Card Electrophysiol Clin. 2020 Jun;12(2):233-245. doi: 10.1016/j.ccep.2020.01.001. PMID: 32451107.

62: Gianni C, Rivera D, Burkhardt JD, Pollard B, Gardner E, Maguire P, Zei PC, Natale A, Al-Ahmad A. Stereotactic arrhythmia radioablation for refractory scar- related ventricular tachycardia. Heart Rhythm. 2020 Aug;17(8):1241-1248. doi: 10.1016/j.hrthm.2020.02.036. Epub 2020 Mar 6. PMID: 32151737.

63: Roca-Luque I, Van Breukelen A, Alarcon F, Garre P, Tolosana JM, Borras R, Sanchez P, Zaraket F, Doltra A, Ortiz-Perez JT, Prat-Gonzalez S, Perea RJ, Guasch E, Arbelo E, Berruezo A, Sitges M, Brugada J, Mont L. Ventricular scar channel entrances identified by new wideband cardiac magnetic resonance sequence to guide ventricular tachycardia ablation in patients with cardiac defibrillators. Europace. 2020 Apr 1;22(4):598-606. doi: 10.1093/europace/euaa021. PMID: 32101605.

64: Kubala M, Xie S, Santangeli P, Garcia FC, Supple GE, Schaller RD, Liang JJ, Pathak RK, Zado ES, Tschabrunn C, Arkles J, Callans DJ, Marchlinski FE. Analysis of local ventricular repolarization using unipolar recordings in patients with arrhythmogenic right ventricular cardiomyopathy. J Interv Card Electrophysiol. 2020 Mar;57(2):261-270. doi: 10.1007/s10840-019-00594-z. Epub 2019 Aug 23. PMID: 31440875.

65: Aktas MK, Huang DT. Mapping and ablation of ventricular tachycardia 36 years after a Pennsylvania peel. HeartRhythm Case Rep. 2020 Apr 13;6(7):431-433. doi: 10.1016/j.hrcr.2020.04.002. PMID: 32695594; PMCID: PMC7361175.

66: Troisi F, Quadrini F, Di Monaco A, Vitulano N, Caruso R, Guida P, Langialonga T, Grimaldi M. Electroanatomic guidance versus conventional fluoroscopy during transseptal puncture for atrial fibrillation ablation. J Cardiovasc Electrophysiol. 2020 Jul 23. doi: 10.1111/jce.14683. Epub ahead of print. PMID: 32700436.

67: Scaglione M, Ebrille E, Caponi D, Battaglia A, Di Donna P, Anselmino M, Peyracchia M, Mazzucchi P, Cerrato N, Ferraris F, Castagno D, Lamberti F, Gaita F. Zero-fluoroscopy atrial fibrillation ablation in the presence of a patent foramen ovale: a multicentre experience. J Cardiovasc Med (Hagerstown). 2020 Apr;21(4):292-298. doi: 10.2459/JCM.0000000000000943. PMID: 32068573.

68: Tomasi C, Dal Monte A, Argnani MS, Corsi C, Giannotti F, Severi S, Rubboli A. Impedance mapping with constant contact force on 3D electroanatomic map to characterize tissues at pulmonary veno-atrial junction. J Interv Card Electrophysiol. 2020 Aug 4. doi: 10.1007/s10840-020-00845-4. Epub ahead of print. PMID: 32749567.

69: Xhaët O, Deceuninck O, Robaye B, Dormal F, Collet B, Godeaux V, Huys F, Ballant E, Gourdin M, Blommaert D. A circular mapping catheter is not mandatory for isolating pulmonary veins during paroxysmal atrial fibrillation ablation with radiofrequency. J Interv Card Electrophysiol. 2020 Oct 19. doi: 10.1007/s10840-020-00895-8. Epub ahead of print. PMID: 33074448.

70: Nazarian S, Markman TM. Mapping Endocardial-Epicardial Dissociation: Significance for Atrial Fibrillation Ablation. JACC Clin Electrophysiol. 2020 Jul;6(7):846-848. doi: 10.1016/j.jacep.2020.04.026. PMID: 32703567.

71: Muser D, Santangeli P. Ventricular Arrhythmias in Myocarditis: Prognostic Role of Electroanatomic Voltage Mapping. JACC Clin Electrophysiol. 2020 May;6(5):583-585. doi: 10.1016/j.jacep.2020.01.008. PMID: 32439045.

72: Guttman MA, Tao S, Fink S, Tunin R, Schmidt EJ, Herzka DA, Halperin HR, Kolandaivelu A. Acute enhancement of necrotic radio-frequency ablation lesions in left atrium and pulmonary vein ostia in swine model with non-contrast- enhanced T_1-weighted MRI. Magn Reson Med. 2020 Apr;83(4):1368-1379. doi: 10.1002/mrm.28001. Epub 2019 Sep 30. PMID: 31565818; PMCID: PMC6949368.

73: Athar AM, Nabors CC, Dhaduk K, Yandrapalli S, Jain A, Moorthy CR, Halperin EC, Iwai S, Frishman WH, Jacobson J. Noninvasive Radioablation of Ventricular Tachycardia. Cardiol Rev. 2020 Nov/Dec;28(6):283-290. doi: 10.1097/CRD.0000000000000321. PMID: 33017363.

74: Balli S, Kucuk M. Transcatheter ablation using near-zero fluoroscopy in children with focal atrial tachycardia: a single-centre experience. Cardiol Young. 2020 Sep;30(9):1266-1272. doi: 10.1017/S1047951120001973. Epub 2020 Jul 20. PMID: 32684196.

75: Gizatulina TP, Martyanova LU, Petelina TI, Zueva EV, Shirokov NE, Kolunin GV, Belonogov DV, Gorbatenko EA. [The association of growth differentiation factor 15 (GDF-15) level with extent of left atrial fibrosis in patients with nonvalvular atrial fibrillation]. Kardiologiia. 2020 Oct 14;60(9):22-29. Russian. doi: 10.18087/cardio.2020.9.n1144. PMID: 33131471.

76: Obeng-Gyimah E, Nazarian S. Advancements in Imaging for Atrial Fibrillation Ablation: Is There a Potential to Improve Procedural Outcomes? J Innov Card Rhythm Manag. 2020 Jul 15;11(7):4172-4178. doi: 10.19102/icrm.2020.110701. PMID: 32724708; PMCID: PMC7377648.

77: Kushnir A, Pallister KH, Chaudhary SB, Cevasco M, Naka Y, Saluja D. High- density substrate and activation mapping of epicardial ventricular tachycardia during left ventricular assist device implant. HeartRhythm Case Rep. 2020 Jul 4;6(10):690-693. doi: 10.1016/j.hrcr.2020.06.023. PMID: 33101933; PMCID: PMC7573345.

78: Loh P, van Es R, Groen MHA, Neven K, Kassenberg W, Wittkampf FHM, Doevendans PA. Pulmonary Vein Isolation With Single Pulse Irreversible Electroporation: A First in Human Study in 10 Patients With Atrial Fibrillation. Circ Arrhythm Electrophysiol. 2020 Oct;13(10):e008192. doi: 10.1161/CIRCEP.119.008192. Epub 2020 Sep 8. PMID: 32898450.

79: Jiang CX, Long DY, Li MM, Sang CH, Tang RB, Wang W, Li SN, Guo XY, Bai R, Du X, Dong JZ, Ma CS. Evidence of 2 conduction exits of the moderator band: Findings from activation and pace mapping study. Heart Rhythm. 2020 Nov;17(11):1856-1863. doi: 10.1016/j.hrthm.2020.06.014. Epub 2020 Jun 18. PMID: 32562870.

80: Ebert M, Wijnmaalen AP, de Riva M, Trines SA, Androulakis AFA, Glashan CA, Schalij MJ, Peter van Tintelen J, Jongbloed JDH, Zeppenfeld K. Prevalence and Prognostic Impact of Pathogenic Variants in Patients With Dilated Cardiomyopathy Referred for Ventricular Tachycardia Ablation. JACC Clin Electrophysiol. 2020 Sep;6(9):1103-1114. doi: 10.1016/j.jacep.2020.04.025. Epub 2020 Jul 29. PMID: 32972544.

81: Hong KL, Borges J, Glover B. Catheter ablation for the management of atrial fibrillation: current technical perspectives. Open Heart. 2020 May;7(1):e001207. doi: 10.1136/openhrt-2019-001207. PMID: 32393656; PMCID: PMC7223467.

82: Straube F, Dorwarth U, Hartl S, Brueck B, Pongratz J, Kosmalla A, Wankerl M, Hoffmann E. Benefit of ultra-high-density mapping-guided radiofrequency reablation in pulmonary vein isolation non-

responders after initial cryoballoon procedure. Europace. 2020 Jun 1;22(6):906-915. doi: 10.1093/europace/euaa055. PMID: 32361733.

83: Anter E, Neužil P, Rackauskas G, Peichl P, Aidietis A, Kautzner J, Nakagawa H, Jackman WM, Natale A, Reddy VY. A Lattice-Tip Temperature-Controlled Radiofrequency Ablation Catheter for Wide Thermal Lesions: First-in-Human Experience With Atrial Fibrillation. JACC Clin Electrophysiol. 2020 May;6(5):507-519. doi: 10.1016/j.jacep.2019.12.015. Epub 2020 Feb 26. PMID: 32439034.

84: Cronin EM, Bogun FM, Maury P, Peichl P, Chen M, Namboodiri N, Aguinaga L, Leite LR, Al-Khatib SM, Anter E, Berruezo A, Callans DJ, Chung MK, Cuculich P, d'Avila A, Deal BJ, Della Bella P, Deneke T, Dickfeld TM, Hadid C, Haqqani HM, Kay GN, Latchamsetty R, Marchlinski F, Miller JM, Nogami A, Patel AR, Pathak RK, Saenz Morales LC, Santangeli P, Sapp JL Jr, Sarkozy A, Soejima K, Stevenson WG, Tedrow UB, Tzou WS, Varma N, Zeppenfeld K. 2019 HRS/EHRA/APHRS/LAHRS expert consensus statement on catheter ablation of ventricular arrhythmias: Executive summary. J Interv Card Electrophysiol. 2020 Oct;59(1):81-133. doi: 10.1007/s10840-019-00664-2. PMID: 31960344; PMCID: PMC7508755.

85: Adragão P, Matos D, Costa FM, Carmo P, Cavaco D, Rodrigues G, Carmo J, Morgado F, Mendes M. A new electrophysiologic triad for identification and localization of the critical isthmus in atrial flutter. Rev Port Cardiol. 2020 Jun;39(6):309-314. English, Portuguese. doi: 10.1016/j.repc.2020.06.009. Epub 2020 Jul 9. PMID: 32654877.

86: Kubala M, Tschabrunn C, Marchlinski DF, Marchlinski FE. Overcoming challenges in the management of arrhythmogenic right ventricular cardiomyopathy. Kardiol Pol. 2020 May 25;78(5):386-395. doi: 10.33963/KP.15374. Epub 2020 May 19. PMID: 32431133.

87: Zhang Y, Li XM. Pre-excitation cardiac problems in children: recognition and treatment. Eur J Pediatr. 2020 Aug;179(8):1197-1204. doi: 10.1007/s00431-020-03701-9. Epub 2020 Jun 11. PMID: 32529398.

88: Gizatulina TP, Martyanova LU, Pavlov AV, Shirokov NE, Kolunin GV, Belonogov DV, Gorbatenko EA. [Predictors of Left Atrial Severe Fibrosis in Patients with Nonvalvular Atrial Fibrillation]. Kardiologiia. 2020 Mar 5;60(2):47-53. Russian. doi: 10.18087/cardio.2020.2.n850. PMID: 32345198.

89: Barbhaiya CR, Kogan EV, Jankelson L, Knotts RJ, Spinelli M, Bernstein S, Park D, Aizer A, Chinitz LA, Holmes D. Esophageal temperature dynamics during high-power short-duration posterior wall ablation. Heart Rhythm. 2020 May;17(5 Pt A):721-727. doi: 10.1016/j.hrthm.2020.01.014. Epub 2020 Jan 21. PMID: 31978595.

90: Tsujioka S, Nozoe M, Kawano Y, Suematsu N, Kubota T. Successful Catheter Ablation for Multiple Atrial Arrhythmias in a Patient with Situs Inversus Totalis. Intern Med. 2020 Oct 7. doi: 10.2169/internalmedicine.5361-20. Epub ahead of print. PMID: 33028769.

91: Balli S, Kucuk M, Epçaçan S. Transcatheter radiofrequency ablation using near-zero fluoroscopy in children with fascicular ventricular tachycardia: a single-centre experience. Cardiol Young. 2020 Jun;30(6):779-784. doi: 10.1017/S104795112000102X. Epub 2020 May 8. PMID: 32383414.

92: Pelargonio G, Pinnacchio G, Narducci ML, Pieroni M, Perna F, Bencardino G, Comerci G, Dello Russo A, Casella M, Bartoletti S, Russo E, Crea F. Long-Term Arrhythmic Risk Assessment in Biopsy-Proven Myocarditis. JACC Clin Electrophysiol. 2020 May;6(5):574-582. doi: 10.1016/j.jacep.2019.12.010. Epub 2020 Feb 26. PMID: 32439044.

93: Santoro A, Baiocchi C, Lumia G, Zacà V, Romano A, Spera L, Stricagnoli M, Falciani F, Valente S, Gaspardone A, Mondillo S, Lamberti F. Detection of oesophageal course during left atrial catheter

ablation. Indian Pacing Electrophysiol J. 2020 Jun 27:S0972-6292(20)30062-0. doi: 10.1016/j.ipej.2020.06.003. Epub ahead of print. PMID: 32599079.

94: Nakatani Y, Sakamoto T, Yamaguchi Y, Tsujino Y, Kinugawa K. Epicardial adipose tissue affects the efficacy of left atrial posterior wall isolation for persistent atrial fibrillation. J Arrhythm. 2020 May 16;36(4):652-659. doi: 10.1002/joa3.12359. PMID: 32782636; PMCID: PMC7411190.

95: Kharazi AL, Hernandez FV, Mounsey JP, Kiser AC. Endocardial and Epicardial Rhythmia HDx™ Mapping Verifies Surgical Cox Maze IV Lesion Pattern. J Innov Card Rhythm Manag. 2020 Jan 15;11(1):3969-3974. doi: 10.19102/icrm.2020.110104. PMID: 32368366; PMCID: PMC7192144.

96: van Loon G, Van Steenkiste G, Vera L, Decloedt A. Catheter-based electrical interventions to study, diagnose and treat arrhythmias in horses: From refractory period to electro-anatomical mapping. Vet J. 2020 Sep;263:105519. doi: 10.1016/j.tvjl.2020.105519. Epub 2020 Jul 24. PMID: 32928488.

97: Mizobuchi M, Yamashita T, Kobayashi T, Nakamura S. Unrecognized left atrial activation patterns of Marshall bundle-related atrial tachycardia following atrial fibrillation ablation. HeartRhythm Case Rep. 2020 May 16;6(8):543-546. doi: 10.1016/j.hrcr.2020.05.005. PMID: 32817838; PMCID: PMC7424301.

98: Hoogendoorn JC, Sramko M, Venlet J, Siontis KC, Kumar S, Singh R, Nakajima I, Piers SRD, de Riva Silva M, Glashan CA, Crawford T, Tedrow UB, Stevenson WG, Bogun F, Zeppenfeld K. Electroanatomical Voltage Mapping to Distinguish Right- Sided Cardiac Sarcoidosis From Arrhythmogenic Right Ventricular Cardiomyopathy. JACC Clin Electrophysiol. 2020 Jun;6(6):696-707. doi: 10.1016/j.jacep.2020.02.008. Epub 2020 Apr 29. PMID: 32553221.

99: Žižek D, Antolič B, Prolič Kalinšek T, Štublar J, Kajdič N, Jelenc M, Jan M. Intracardiac echocardiography-guided transseptal puncture for fluoroless catheter ablation of left-sided tachycardias. J Interv Card Electrophysiol. 2020 Aug 28. doi: 10.1007/s10840-020-00858-z. Epub ahead of print. PMID: 32860178.

100: Al-Kaisey AM, Parameswaran R, Joseph SA, Kistler PM, Morton JB, Kalman JM. Extensive right atrial free wall low-voltage zone as the substrate for atrial fibrillation: successful ablation by scar homogenization. Europace. 2020 Nov 3:euaa233. doi: 10.1093/europace/euaa233. Epub ahead of print. PMID: 33141888.

101: Reddy VY, Neužil P, Peichl P, Rackauskas G, Anter E, Petru J, Funasako M, Minami K, Aidietis A, Marinskis G, Natale A, Nakagawa H, Jackman WM, Kautzner J. A Lattice-Tip Temperature-Controlled Radiofrequency Ablation Catheter: Durability of Pulmonary Vein Isolation and Linear Lesion Block. JACC Clin Electrophysiol. 2020 Jun;6(6):623-635. doi: 10.1016/j.jacep.2020.01.002. Epub 2020 Jan 24. PMID: 32553211.

102: Silva MA, Futuro GMC, Merçon ES, Vasconcelos D, Agrizzi RS, Elias Neto J, Kuniyoshi R. Safety of Catheter Ablation of Atrial Fibrillation Under Uninterrupted Rivaroxaban Use. Arq Bras Cardiol. 2020 Mar;114(3):435-442. English, Portuguese. doi: 10.36660/abc.20180386. PMID: 32049156.

103: Guarguagli S, Cazzoli I, Kempny A, Gatzoulis MA, Ernst S. Initial Experience Using the Radiofrequency Needle Visualization on the Electroanatomical Mapping System for Transseptal Puncture. Cardiol Res Pract. 2020 Jun 20;2020:5420909. doi: 10.1155/2020/5420909. PMID: 32655947; PMCID: PMC7322610.

104: Quesada A, Cózar R, Anguera I; Spanish Catheter Ablation Registry collaborators. Spanish Catheter Ablation Registry. 19th Official Report of the Heart Rhythm Association of the Spanish Society of Cardiology (2019). Rev Esp Cardiol (Engl Ed). 2020 Nov 2:S1885-5857(20)30430-8. English, Spanish. doi: 10.1016/j.rec.2020.08.022. Epub ahead of print. PMID: 33153956.

105: Della Bella P, Peretto G, Paglino G, Bisceglia C, Radinovic A, Sala S, Baratto F, Limite LR, Cireddu M, Marzi A, D'Angelo G, Vergara P, Gulletta S, Mazzone P, Frontera A. Bipolar radiofrequency ablation for ventricular tachycardias originating from the interventricular septum: Safety and efficacy in a pilot cohort study. Heart Rhythm. 2020 Jun 26:S1547-5271(20)30624-X. doi: 10.1016/j.hrthm.2020.06.025. Epub ahead of print. PMID: 32599177.

106: Salam T, Wilson L, Bohannan S, Morin M. Safety and Effectiveness of a Novel Fluoroless Transseptal Puncture Technique for Lead-free Catheter Ablation: A Case Series. J Innov Card Rhythm Manag. 2020 Apr 15;11(4):4079-4085. doi: 10.19102/icrm.2020.110405. PMID: 32368383; PMCID: PMC7192152.

107: Chu H, Du X, Shen C, He B, Feng M, Liu J, Fu G, Wang B. Left atrial appendage closure with zero fluoroscopic exposure via intracardiac echocardiographic guidance. J Formos Med Assoc. 2020 Nov;119(11):1586-1592. doi: 10.1016/j.jfma.2020.07.021. Epub 2020 Jul 20. PMID: 32703696.

108: Venlet J, Tao Q, de Graaf MA, Glashan CA, de Riva Silva M, van der Geest RJ, Scholte AJ, Piers SRD, Zeppenfeld K. RV Tissue Heterogeneity on CT: A Novel Tool to Identify the VT Substrate in ARVC. JACC Clin Electrophysiol. 2020 Sep;6(9):1073-1085. doi: 10.1016/j.jacep.2020.04.029. Epub 2020 May 12. PMID: 32972541.

109: Malaczynska-Rajpold K, Blaszyk K, Kociemba A, Pyda M, Posadzy-Malaczynska A, Grajek S. Islets of heterogeneous myocardium within the scar in cardiac magnetic resonance predict ventricular tachycardia after myocardial infarction. J Cardiovasc Electrophysiol. 2020 Jun;31(6):1452-1461. doi: 10.1111/jce.14461. Epub 2020 Apr 24. PMID: 32227520.

110: Kariki O, Antoniou CK, Mavrogeni S, Gatzoulis KA. Updating the Risk Stratification for Sudden Cardiac Death in Cardiomyopathies: The Evolving Role of Cardiac Magnetic Resonance Imaging. An Approach for the Electrophysiologist. Diagnostics (Basel). 2020 Jul 31;10(8):541. doi: 10.3390/diagnostics10080541. PMID: 32751773; PMCID: PMC7460122.

111: Kuo L, Liang JJ, Han Y, Frankel DS, Santangeli P, Callans DJ, Zado ES, Marchlinski FE, Desjardins B, Nazarian S. Association of septal late gadolinium enhancement on cardiac magnetic resonance with ventricular tachycardia ablation targets in nonischemic cardiomyopathy. J Cardiovasc Electrophysiol. 2020 Oct 18. doi: 10.1111/jce.14777. Epub ahead of print. PMID: 33070414.

112: Chaudhry-Waterman N, Kumar V, Karr S, Fitzpatrick A, Cohen MI. Challenge of managing opposing rhythms in a mother and fetus. Pacing Clin Electrophysiol. 2020 Sep 8. doi: 10.1111/pace.14059. Epub ahead of print. PMID: 32896920.

113: Margato R, Tampakis K, Albenque JP, Combes S. Illuminating the Marshall: novel techniques highlighted in an atrial tachycardia case report. Eur Heart J Case Rep. 2020 Aug 3;4(4):1-5. doi: 10.1093/ehjcr/ytaa229. PMID: 32974439; PMCID: PMC7501895.

114: Yu R, Liu N, Lu J, Zhao X, Hu Y, Zhang J, Xu F, Tang R, Bai R, Akar JG, Dong J, Ma C. 3-Dimensional Transseptal Puncture Based on Electrographic Characteristics of Fossa Ovalis: A Fluoroscopy-Free and Echocardiography-Free Method. JACC Cardiovasc Interv. 2020 May 25;13(10):1223-1232. doi: 10.1016/j.jcin.2020.03.015. PMID: 32438994.

115: Chin R, Hayase J, Hu P, Cao M, Deng J, Ajijola O, Do D, Vaseghi M, Buch E, Khakpour H, Fujimura O, Krokhaleva Y, Macias C, Sorg J, Gima J, Pavez G, Boyle NG, Steinberg M, Shivkumar K, Bradfield JS. Non-invasive stereotactic body radiation therapy for refractory ventricular arrhythmias: an institutional experience. J Interv Card Electrophysiol. 2020 Aug 15. doi: 10.1007/s10840-020-00849-0. Epub ahead of print. PMID: 32803639.

116: Hoogendoorn JC, Ninaber MK, Piers SRD, de Riva M, Grauss RW, Bogun FM, Zeppenfeld K. The harm of delayed diagnosis of arrhythmogenic cardiac sarcoidosis: a case series. Europace. 2020 Sep 1;22(9):1376-1383. doi: 10.1093/europace/euaa115. PMID: 32898252; PMCID: PMC7478317.

117: Herczeg S, Galvin J, Keaney JJ, Keelan E, Byrne R, Howard C, Geller L, Szeplaki G. The Value of Voltage Histogram Analysis Derived Right Atrial Scar Burden in the Prediction of Left Atrial Scar Burden. Cardiol Res Pract. 2020 Aug 13;2020:3981684. doi: 10.1155/2020/3981684. PMID: 32855820; PMCID: PMC7442993.

118: Quesada A, Cózar R, Anguera I; en representación de los colaboradores del Registro Español de Ablación con Catéter. Registro Español de Ablación con Catéter. XIX Informe Oficial de la Asociación del Ritmo Cardiaco de la Sociedad Española de Cardiología (2019) [Spanish Catheter Ablation Registry. 19th Official Report of the Heart Rhythm Association of the Spanish Society of Cardiology (2019)]. Rev Esp Cardiol. 2020 Sep 23. Spanish. doi: 10.1016/j.recesp.2020.08.005. Epub ahead of print. PMID: 32982011; PMCID: PMC7509535.

119: Zhang G, Cheng L, Liang Z, Zhang J, Dong R, Hang F, Wang X, Wang Z, Zhao L, Wang Z, Wu Y. Zero-fluoroscopy transseptal puncture guided by right atrial electroanatomical mapping combined with intracardiac echocardiography: A single-center experience. Clin Cardiol. 2020 Sep;43(9):1009-1016. doi: 10.1002/clc.23401. Epub 2020 Jun 7. PMID: 32506504; PMCID: PMC7462191.

120: Gil-Ortega I, Serna-Berná A, Trujillo-Santos AJ, Ramos-Amores D, Garrido- Corro B, Cortez-Salazar RJ, Archondo-Arce TG, Castillo-Moreno JA. Non fluoroscopic ablation of different arrhythmic structures in an electrophysiology unit. Assessment of efficiency and security. Ir J Med Sci. 2020 Jun 8. doi: 10.1007/s11845-020-02247-x. Epub ahead of print. PMID: 32514660.

121: Zhou X, Jiang Y, Sohinki D, Liu W, Po SS. Effects of 60-Hertz notch filtering on local abnormal ventricular activities. Heart Rhythm. 2020 Sep 8:S1547-5271(20)30863-8. doi: 10.1016/j.hrthm.2020.08.022. Epub ahead of print. PMID: 32911051.

122: Kaur D, Roukoz H, Shah M, Yalagudri S, Pandurangi U, Chennapragada S, Narasimhan C. Impact of the inflammation on the outcomes of catheter ablation of drug-refractory ventricular tachycardia in cardiac sarcoidosis. J Cardiovasc Electrophysiol. 2020 Mar;31(3):612-620. doi: 10.1111/jce.14341. Epub 2020 Jan 27. PMID: 31916658.

123: Varma N, Rizzo R, Wisnoskey B. An atrial fibrillation rotor, mapped conventionally. J Cardiovasc Electrophysiol. 2020 Feb;31(2):544-546. doi: 10.1111/jce.14329. Epub 2020 Jan 13. PMID: 31908076.

124: Muser D, Santangeli P, Castro SA, Liang JJ, Enriquez A, Liuba I, Magnani S, Garcia FC, Arkles J, Supple GG, Lin D, Schaller RD, Kumareswaran R, Zado E, Tschabrunn CM, Dixit S, Frankel DS, Callans DJ, Marchlinski FE. Collateral injury of the conduction system during catheter ablation of septal substrate in nonischemic cardiomyopathy. J Cardiovasc Electrophysiol. 2020 Jul;31(7):1726-1739. doi: 10.1111/jce.14498. Epub 2020 May 5. PMID: 32298038.

125: Hayashi Y, Shimeno K, Nakatsuji K, Naruko T. What is the mechanism of narrow paced QRS duration during left bundle branch area pacing? A case report. Eur Heart J Case Rep. 2020 Aug 12;4(4):1-5. doi: 10.1093/ehjcr/ytaa239. PMID: 32974479; PMCID: PMC7501936.

126: Nevvazhay T, Zeppenfeld K, Brouwer C, Hazekamp M. Intraoperative cryoablation in late pulmonary valve replacement for tetralogy of Fallot. Interact Cardiovasc Thorac Surg. 2020 May 1;30(5):780-782. doi: 10.1093/icvts/ivaa013. PMID: 32298427.

127: Houmsse M, Daoud EG, Joseph M, Weiss R, Essandoh M. Evaluation of a novel esophageal retractor utilizing vacuum suction and mechanical force for deviating the esophagus. J Cardiovasc

Electrophysiol. 2020 Jul;31(7):1661-1669. doi: 10.1111/jce.14529. Epub 2020 May 15. PMID: 32369243.

128: Aksan G. Catheter Ablation of Left Ventricular Summit Arrhythmia in a Patient with Critical Coronary Artery Stenosis: A Sequential Approach. J Innov Card Rhythm Manag. 2020 Oct 15;11(10):4266-4271. doi: 10.19102/icrm.2020.111004. PMID: 33123415; PMCID: PMC7588238.

129: Kashou AH, DeSimone CV, Asirvatham SJ, Kapa S. Left atrial dissection as a trigger for recurrent atrial fibrillation. HeartRhythm Case Rep. 2020 Mar 6;6(6):329-333. doi: 10.1016/j.hrcr.2020.02.011. PMID: 32577388; PMCID: PMC7300347.

130: Nair GM, Nery PB. Complex Atrial Tachycardias: Is Technology the Answer or Should the Focus Be on Prevention? JACC Clin Electrophysiol. 2020 Jul;6(7):827-829. doi: 10.1016/j.jacep.2020.04.018. PMID: 32703565.

131: Hohmann S, Henkenberens C, Zormpas C, Christiansen H, Bauersachs J, Duncker D, Veltmann C. A novel open-source software-based high-precision workflow for target definition in cardiac radioablation. J Cardiovasc Electrophysiol. 2020 Jul 10. doi: 10.1111/jce.14660. Epub ahead of print. PMID: 32648343.

132: Saleh M, Coleman KM, Vaishnav AS, Shein J, Makker P, Skipitaris N, Mountantonakis SE. Intracardiac echocardiography guided nonocclusive balloon cryothermal applications to achieve antral isolation during pulmonary vein isolation. J Interv Card Electrophysiol. 2020 Oct 27. doi: 10.1007/s10840-020-00905-9. Epub ahead of print. PMID: 33106958.

133: Di Marco A, Ruiz-Cueto M, Salazar-Mendiguchía J, Claver E, Roura G, Dallaglio PD, Anguera I. Genotype-phenotype correlation of LMNA variants involving the Arg541 residue: a case report with multimodality imaging and literature review. ESC Heart Fail. 2020 Oct;7(5):3169-3173. doi: 10.1002/ehf2.12776. Epub 2020 Jul 15. PMID: 32667740; PMCID: PMC7524116.

134: Montgomery JA. Focal arrhythmia ablation with multipolar mapping: Does it still make sense to stay off-grid? J Cardiovasc Electrophysiol. 2020 Jun 24. doi: 10.1111/jce.14634. Epub ahead of print. PMID: 32583626.

135: Muser D, Santangeli P. Detection of Intramural Excitable Substrate With Frequency Analysis of Unipolar Electrograms: A Novel Application of Unipolar Electrogram Mapping. JACC Clin Electrophysiol. 2020 Jul;6(7):770-772. doi: 10.1016/j.jacep.2020.04.004. PMID: 32703557.

136: Nishimura T, Kannan A, Tung R. Reentrant para-Hisian ventricular tachycardia eliminated from the noncoronary cusp: Importance of regional anatomy for vantage-point ablation. J Cardiovasc Electrophysiol. 2020 Apr;31(4):968-971. doi: 10.1111/jce.14401. Epub 2020 Mar 4. PMID: 32077542.
