# Supplementary material for: OpenEP: A Cross-Platform Electroanatomic Mapping Data Format and Analysis Platform for Electrophysiology Research
Source: Front Physiol. 2021 Feb 26;12:646023. doi: 10.3389/fphys.2021.646023 (PMC7952326; doi:10.3389/fphys.2021.646023)
Supplement: Supplementary file 3 [file Data_Sheet_3.PDF]

Supplementary Material

Supplementary Table 2: Available parameter/value pairs for `plotOpenEPEgms()`

Parameter	Values	Description
`'iEgm'`	`Integer`	The values of `iEgm` index into the fields in `userdata.electric` and the corresponding electrograms are drawn.
`'range'`	`'window'` or `'all'`	The value `'window'` (default) specifies that only the electrogram within the window of interest is plotted. The value 'all' specifies that the entire electrogram is plotted.
`'buffer'`	`Double`	If range is `'window'` then the value of buffer is plotted either side of the reference window of interest. The default value of `'buffer'` is 50.
`'egmtype'`	`'bip'`, `'uni'`, or `'bip-uni'`	The value of `'egmtype'` specifies whether to plot the bipolar electrogram alone, the associated unipolar electrograms or both.
`'reference'`	`'on'` or `'off'`	The value of `'reference'` specifies whether to plot the electrogram recorded from the reference channel
