# Supplementary material for: OpenEP: A Cross-Platform Electroanatomic Mapping Data Format and Analysis Platform for Electrophysiology Research
Source: Front Physiol. 2021 Feb 26;12:646023. doi: 10.3389/fphys.2021.646023 (PMC7952326; doi:10.3389/fphys.2021.646023)
Supplement: Supplementary file 7 [file Data_Sheet_7.PDF]

Supplementary Material

***Supplementary Table 1: Available OpenEP Methods for Calculating Total Activation Time**. Entries in the Value column refer to the value to set for the parameter* `'method'` *in the OpenEP* `getTotalActivationTime()` *function.*

**Method**	**'Method' parameter value**	**Description**
Point-based TAT	`'ptbased'`	Calculates the difference in activation time between the earliest and latest activation time mapping points exported by the clinical system.
Point-based TAT using the percentile method	`'ptbasedprct'`	First calculates the 0-2.5th percentile and the 97.5-100th percentile mapping times on the exported electrogram annotations, then calculates the difference between the means of these sets of activation times.
Clinical local activation time map-based TAT	`'clinmap'`	Calculates the difference between the earliest and latest activation times on the local activation time map created by the clinical mapping system.
Clinical local activation time map-based TAT using the percentile method	`'clinmapprct'`	First calculates the 0-2.5th percentile and the 97.5-100th percentile mapping times on the clinical local activation time map, then calculates the difference between the means of these sets of activation times.
OpenEP interpolated local activation time map-based TAT	`'openepmap'`	Calculates the difference between the earliest and latest activation times on the local activation time map created by OpenEP from the exported electrogram annotations.
OpenEP interpolated local activation time map-based TAT using the percentile method	`'openepmapprct'`	First calculates the 0-2.5th percentile and the 97.5-100th percentile mapping times on the local activation time map created by OpenEP from the exported electrogram annotations. Then calculates the difference between the means of these sets of activation times.
