# Supplementary material for: OpenEP: A Cross-Platform Electroanatomic Mapping Data Format and Analysis Platform for Electrophysiology Research
Source: Front Physiol. 2021 Feb 26;12:646023. doi: 10.3389/fphys.2021.646023 (PMC7952326; doi:10.3389/fphys.2021.646023)
Supplement: Supplementary file 8 [file Table_1.docx]

**Table 1.** **Available OpenEP Functions for Data Processing**. Where parameter-value pairs are to be provided as arguments to a function, the available options for values are shown in a list separated by ‘|’. The OpenEP functions contain internal defaults for all of these parameter-value pairs, with the default value indicated by curly braces ({}).

| **Function** | **Arguments** | **Description** |
| --- | --- | --- |
| **Geometry Functions** | | |
| distanceBetweenPoints(...) | Mandatory arguments:   \| 1 \| userdata \| \| --- \| --- \| \| 2 \| Point 1 \| \| 3 \| Point 2 \|   Parameter-value pairs   \| ‘method’ \| {‘linear’} \| ’geodesic’ \| \| --- \| --- \| \| ‘plot’ \| {‘false’} \| true \| | Use case  Calculate the distance between two points in a straight line or across the surface of the geometry.  Description  Calculate the distance between two points in a straight line or across the surface of the geometry.  Example function call(s)  d = distanceBetweenPoints(userdata, 1, 2, ’method’, ‘geodesic’, ‘plot’, ‘true’) |
| getAnatomicalStructures(...) | Mandatory input arguments:   \| 1 \| userdata \| \| --- \| --- \|   Input parameter-value pairs   \| ‘plot’ \| {false} \| true \| \| --- \| --- \|   Output arguments   \| FF \| See TriRep/freeBoundary, cell array \| \| --- \| --- \| \| l \| Array of lengths (perimeters) of each anatomical structure \| \| a \| Array of areas of each anatomical structure \| \| tr \| Cell array of triangulations of each anatomical structure \| | Use case  Returns the free boundaries (anatomical structures) described in userdata.  Description  getAnatomicalStructures.m identifies all the anatomical structures of a given data set. Anatomical structures are boundary regions that have been added to an anatomical model in the clinical mapping system. For example, with respect of left atrial ablation, anatomical structures may represent the pulmonary vein ostia, mitral valve annulus or left atrial appendage ostium.  Example function call(s)  [FF, l, a, tr{i}] = getAnatomicalStructures(userdata, 'plot', false); |
| getArea(...) | Mandatory input arguments:   \| 1 \| userdata \| \| --- \| --- \|   Input parameter-value pairs   \| ‘method’ \| {‘nofill’} \| ‘fill’ \| \| --- \| --- \|   Output arguments   \| area \| The surface area (cm^2^) \| \| --- \| --- \| | Use case  Returns the surface area of an anatomical model.  Description  getArea.m Returns the surface area of an anatomical model. The anatomical model can first be closed (filling any holes) by specifying the 'method', 'fill' ('nofill' by default).  Example function call(s)  area = getArea( userdata, 'method', 'fill' ) |
| getCentreOfMass(...) | Mandatory input arguments:   \| 1 \| userdata \| \| --- \| --- \|   Input parameter-value pairs   \| ‘plot’ \| {false} \| true \| \| --- \| --- \|   Output arguments   \| C \| The Cartesian co-ordinates of the centre of mass. \| \| --- \| --- \| | Use case  Returns the centre of mass of the anatomical model defined in userdata.  Description  getCenterOfMass.m calculates the centre of mass of the userdata by accessing a closed surface via the OpenEP function getClosedSurface.m before using centroidOfPolyhedron.m to calculate the centre of mass. The  function centroidOfPolyhedron.m was written by Isfandiyar RASHIDZADE, available through the Mathworks FileExchange:  <https://www.mathworks.com/matlabcentral/fileexchange/63614-centroid-of-triangulated-polyhedron>  Example function call(s)  C = getCentreOfMass( userdata, 'plot', true ); |
| getClosedSurface(...) | Mandatory input arguments:   \| 1 \| userdata \| \| --- \| --- \|   Input parameter-value pairs   \| None \|  \| \| --- \| --- \|   Output arguments   \| tr \|  \| \| --- \| --- \| | Use case  Fills all the holes in the userdata surface  Description  getClosedSurface Returns a new surface representation of the anatomical model with all the holes in the mesh filed. Closes the surface by the following algorithm. First, every complete free boundary is identified. Second, the barycentre of the free boundary is identified. Third, a triangulation is created covering this hole. Finally, the additional triangles are added to the TriRep.  Example function call(s)  tr = getClosedSurface( userdata ); |
| getFaces(...) | Mandatory input arguments:   \| 1 \| userdata \| \| --- \| --- \|   Input parameter-value pairs   \| None \|  \| \| --- \| --- \|   Output arguments   \| faces \| All the faces \| \| --- \| --- \| | Use case  Returns the faces referenced by userdata  Description  Returns the faces referenced by userdata  Example function call(s)  faces = getFaces( userdata ); |
| getMesh(...) | Mandatory input arguments:   \| 1 \| Userdata \| \| --- \| --- \|   Input parameter-value pairs   \| ‘type’ \| {‘trirep’} \| ‘triangulation’ \| \| --- \| --- \|   Output arguments   \| tr \| A TriRep, or Triangulation, object \| \| --- \| --- \| | Use case  Returns the triangulation-based mesh from userdata  Description  Returns a face/vertex representation of the anatomical model. Supported data types include instances of the Matlab objects Trirep and Triangulation.  Example function call(s)  [vertices, isVertUsed] = getVertices( userdata ); |
| getVertices(...) | Mandatory input arguments:   \| 1 \| userdata \| \| --- \| --- \|   Input parameter-value pairs   \| None \|  \| \| --- \| --- \|   Output arguments   \| vertices \| All the vertices. \| \| --- \| --- \| \| isVertUsed \| Whether the vertex is referenced by the triangulation. \| | Use case  Returns the vertices referenced by userdata  Description  Returns the vertices referenced by userdata  Example function call(s)  [vertices, isVertUsed] = getVertices( userdata ); |
| getVolume(...) | Mandatory input arguments:   \| 1 \| userdata \| \| --- \| --- \|   Input parameter-value pairs   \| None \|  \| \| --- \| --- \|   Output arguments   \| volume \| The volume, in cm^3^ \| \| --- \| --- \| | Use case  Calculates the volume of the chamber described in userdata  Description  Calculates the volume of the chamber described in userdata  Example function call(s)  volume = getVolume( userdata ); |
| **General Data Functions** | | |
| generateInterpData(...) | Mandatory input arguments:   \| 1 \| userdata \| \| --- \| --- \| \| 2 \| datatype \|   Input parameter-value pairs   \| ‘interMethod’ \| ‘nearest’ \| ‘linear’ \| {‘natural’} \| \| --- \| --- \| \| ‘exterMethod’ \| {‘nearest’} \| ‘linear’ \| ‘none’ \| \| ‘distanceThresh’ \| {10}\|double \|   Output arguments   \| interpData \| The interpolated data \| \| --- \| --- \| | Use case  Performs spatial interpolation of scalar data.  Description  generateInterpData performs spatial interpolation of scalar data. Userdata and datatype are mandatory arguments. Datatype may be one of:   \| 'bip-map' \| bipolar voltage; from the exported voltage values \| \| --- \| --- \| \| 'uni-map' \| unipolar voltage; from the exported voltage values \| \| 'lat-map' \| local activation time; from the annotated electrograms \| \| 'bip-egm' \| bipolar voltage; measured by OpenEP on the egms \| \| 'uni-egm' \| unipolar voltage; measured by OpenEP on the egms \| \| 'lat-egm' \| local activation time; measured by OpenEP on the egms \| \| 'cv’ \| conduction velocity \|   generateInterpData removes any NaN values in data (and their  corresponding location(s) in coords) before calling scatteredInterpolant.m with the interpolation/extrapolation methods specified. Any values greater than distanceThresh are removed.  than distancethresh are removed.Example function call(s)  interpData = generateInterpData(userdata, 'bip-map');  interpData = generateInterpData(userdata, 'lat-map'); |
| getIndexFromCartoPointNumber(...) | Mandatory input arguments:   \| 1 \| userdata \| \| --- \| --- \| \| 2 \| pointNumber \|   Input parameter-value pairs   \| None \|  \| \| --- \| --- \|   Output arguments   \| index \| An index (or array of indices) for referencing into the data fields within userdata.electric \| \| --- \| --- \| | Use case  Finds the index of the mapping point at the point number displayed on the Carto mapping system.  Description  Finds the index of the mapping point at the point number displayed on the Carto mapping system.  Example function call(s)  index = getIndexFromCartoPointNumber(userdata, 1); |
| getNumPts(...) | Mandatory input arguments:   \| 1 \| userdata \| \| --- \| --- \|   Input parameter-value pairs   \| None \|  \| \| --- \| --- \|   Output arguments   \| numpts \| The number of mapping points \| \| --- \| --- \| | Use case  Returns the number of mapping points available in the OpenEP dataset.  Description  Returns the number of mapping points available in the OpenEP dataset.  Example function call(s)  numpts = getNumPts(userdata); |
| getSurfaceData(...) | Mandatory input arguments:   \| 1 \| userdata \| \| --- \| --- \| \| 2 \| datatype \|   Input parameter-value pairs   \| None \|  \| \| --- \| --- \|   Output arguments   \| data \| The returned surface mapping data \| \| --- \| --- \| | Use case  Returns surface mapping data from userdata  Description  Returns surface mapping data from userdata. Data type is specified by the ‘datatype’ argument:   \| 'act' \| Activation time \| \| --- \| --- \| \| 'bip' \| Bipolar voltage \| \| 'uni' \| Unipolar voltage \| \| 'imp' \| Impedance \| \| 'frc' \| Force \|   Example function call(s)  data = getSurfaceData(userdata, 'bip'); |
| openEP2VTK(...) | Mandatory input arguments:   \| 1 \| userdata \| \| --- \| --- \|   Input parameter-value pairs   \| ‘datatype’ \| {‘bip’} \| ‘uni’ \| ‘lat’ \| \| --- \| --- \| \| ‘method’ \| {‘map’} \| ‘egm’ \| \| ‘outputfile’ \| {[]} \| string \| ‘openfile’ \|   Output arguments   \| Path2VTKfile \| The path to the file that was written \| \| --- \| --- \| | Use case  Converts the OpenEP format to VTK format.  Description  Converts between OpenEP format and VTK format. This function takes map data and writes it to the VTK file, or if 'method' is set to 'egm' it first uses generateInterpData.m to create interpolated data.  Example function call(s)  % path2VTKfile = openEP2VTK(userdata, 'datatype', 'lat', 'outputfile', 'openfile'); |
| **Activation Data** | | |
| cvHistogram(...) | Mandatory input arguments:   \| 1 \| userdata \| \| --- \| --- \|   Input parameter-value pairs   \| ‘limits’ \| {[0 5]} \| array \| \| --- \| --- \| \| ‘binwidth’ \| {0.1} \| double \|   Output arguments   \| None \|  \| \| --- \| --- \| | Use case  Draws a conduction velocity histogram  Description  cvHistogram.m displays a histogram of conduction velocities. Limits are set to exclude non-physiological conduction velocities  Example function call(s)  cvHistogram( userdata ); |
| getConductionVelocity(...) | Mandatory input arguments:   \| 1 \| userdata \| \| --- \| --- \|   Input parameter-value pairs   \| None \|  \| \| --- \| --- \|   Output arguments   \| cvdata \| The conduction velocities, in m/s \| \| --- \| --- \| | Use case  Returns the conduction velocity map of the chamber  Description  getConductionVelocity.m Calculate conduction velocities by calculating gradients of interpolated local activation times. getConductionVelocity.m makes use of a modified version of "Scattered Data Interpolation and Approximation using Radial Base Functions" available from the Matlab FileExchange: Alex Chirokov (2020). Scattered Data Interpolation and Approximation using Radial Base Functions (<https://www.mathworks.com/matlabcentral/fileexchange/10056-scattered-data-interpolation-and-approximation-using-radial-base-functions>), MATLAB Central File Exchange. Retrieved November 24, 2020.  Example function call(s)  cvdata = getConductionVelocity( userdata ); |
| getEarliesActivationSite(...) | Mandatory input arguments:   \| 1 \| userdata \| \| --- \| --- \|   Input parameter-value pairs   \| ‘method’ \| {'ptbased'} \| 'ptbasedprct' \| 'clinmap' \| 'clinmapprct' \| 'openepmap' \| 'openmapprct' \| \| --- \| --- \| \| ‘prct’ \| {2.5} \| double \|   Output arguments   \| X \| Cartesian co-ordinates of the earliest activation site. For map-based methods (i.e. ‘clinmap’, ‘clinmapprct’, ‘openepmap’ and ‘openepmapprct’), X is identical to surfX. \| \| --- \| --- \| \| surfX \| The surface projection of the earliest activation site \| \| iPoint \| The closest mapping point to the earliest activation site. For point-based methods (i.e. ‘clinmap’, ‘clinmapprct’, ‘openepmap’, ‘openepmapprct’), iPoint indexes into userdata.surface.triRep.X. For percentile methods (i.e. ‘ptbasedprct’, ‘clinmapprct’ or ‘openepmapprct’) iPoint returns all the points that were identified within the relevant percentile. \| \| t \| The calculated earliest activation time, relative to the reference annotation. \| | Use case  Returns the earliest activation site.  Description  By identifying the latest activating site, this function can be used, for example, to identify the site in the chamber closest to the pacing site. Several alternative methods are provided for calculating the earliest activation site, specified by setting the 'method' parameter-value pair to one of the following options:  'ptbased' - Calculates the earliest activation time using the  mapping points exported by the clinical system.  'ptbasedprct'- Calculates the 0-2.5th percentile mapping  times on the exported electrogram annotations, then  calculates the mean of this set of activation times.  'clinmap' - Calculates the earliest activation time on the local  activation time map created by the clinical mapping  system  'clinmapprct'- First calculates the 0-2.5th percentile mapping  times on the clinical local activation time map, then  calculates the mean of this set of activation times.  'openepmap' - Calculates the earliest activation time on the local  activation time map created by OpenEP from the  exported electrogram annotations.  'openepmapprct'- First calculates the 0-2.5th percentile  mapping times on the local activation time map created  by OpenEP from the exported electrogram annotations.  then calculates the mean of this set of activation  Example function call(s)  [X, surfX, iPoint, t] = getEarliestActivationSite( userdata ); |
| getLatestActivationSite(...) | Mandatory input arguments:   \| 1 \| userdata \| \| --- \| --- \|   Input parameter-value pairs   \| ‘method’ \| {'ptbased'} \| 'ptbasedprct' \| 'clinmap' \| 'clinmapprct' \| 'openepmap' \| 'openmapprct' \| \| --- \| --- \| \| ‘prct’ \| {2.5} \| double \|   Output arguments   \| X \| Cartesian co-ordinates of the latest activation site. For map-based methods (i.e. ‘clinmap’, ‘clinmapprct’, ‘openepmap’ and ‘openepmapprct’), X is identical to surfX. \| \| --- \| --- \| \| surfX \| The surface projection of the latest activation site \| \| iPoint \| The closest mapping point to the latest activation site. For point-based methods (i.e. ‘clinmap’, ‘clinmapprct’, ‘openepmap’, ‘openepmapprct’), iPoint indexes into userdata.surface.triRep.X. For percentile methods (i.e. ‘ptbasedprct’, ‘clinmapprct’ or ‘openepmapprct’) iPoint returns all the points that were identified within the relevant percentile. \| \| t \| The calculated latest activation time, relative to the reference annotation. \| | Use case  Returns the latest activation site.  Description  By identifying the latest activating site, this function can be used, for example, to calculate the total activation time. Several alternative methods are provided for calculating the latest activation site, specified by setting the 'method' parameter-value pair to one of the following options:  'ptbased' - Calculates the latest activation time using the  mapping points exported by the clinical system.  'ptbasedprct'- Calculates the 97.5-100th percentile mapping  times on the exported electrogram annotations, then  calculates the mean of this set of activation times.  'clinmap' - Calculates the latest activation time on the local  activation time map created by the clinical mapping  system  'clinmapprct'- First calculates the 97.5-100th percentile  mapping times on the clinical local activation time map,  then calculates the mean of this set of activation times.  'openepmap' - Calculates the latest activation time on the local  activation time map created by OpenEP from the  exported electrogram annotations.  'openepmapprct'- First calculates the 97.5-100th percentile  mapping times on the local activation time map created  by OpenEP from the exported electrogram annotations.  Then calculates the mean of this set of activation times.  Example function call(s)  [X, surfX, iPoint, t] = getLatestActivationSite( userdata ); |
| getMappingPointsWithinWoI(...) | Mandatory input arguments:   \| 1 \| userdata \| \| --- \| --- \|   Input parameter-value pairs   \| None \|  \| \| --- \| --- \|   Output arguments   \| iPoint \|  \| \| --- \| --- \| | Use case  Returns the indices of the mapping points with annotated local activation time within the window of interest.  Description  Returns the indices of the mapping points with annotated local activation time within the window of interest.  Example function call(s)  iPoint = getMappingPointsWithinWoI( userdata ); |
| getTotalActivationTime(...) | Mandatory input arguments:   \| 1 \| userdata \| \| --- \| --- \|   Input parameter-value pairs   \| ‘method’ \| {'ptbased'} \| 'ptbasedprct' \| 'clinmap' \| 'clinmapprct' \| 'openepmap' \| 'openmapprct' \| \| --- \| --- \| \| ‘prct’ \| {2.5} \| double \|   Output arguments   \| tat \| The total activation time, in ms \| \| --- \| --- \| | Use case  Returns the total activation time of the chamber  Description  Returns the total activation time of the chamber. Several alternative methods are provided, and specified by setting the 'method' parameter-value pair to one of the following options:  'ptbased' - Calculates the difference in activation time between  the earliest and latest activation time mapping  points exported by the clinical system.  'ptbasedprct'- First calculates the 0-2.5th percentile and  the 97.5-100th percentile mapping times on the  exported electrogram annotations, then calculates  the difference between the means of these sets of  activation times.  'clinmap' - Calculates the difference between the earliest and  latest activation times on the local activation  time map created by the clinical mapping system  'clinmapprct'- First calculates the 0-2.5th percentile and  the 97.5-100th percentile mapping times on the  clinical local activation time map, then calculates  the difference between the means of these sets of  activation times.  'openepmap' - Calculates the difference between the earliest and  latest activation times on the local activation  time map created by OpenEP from the exported  electrogram annotations.  'openepmapprct'- First calculates the 0-2.5th percentile  and the 97.5-100th percentile mapping times on the local  activation time map created by OpenEP from the  exported electrogram annotations. Then calculates  the difference between the means of these sets of  activation times.  Example function call(s)  tat = getTotalActivationTime( userdata ); |
| **Voltage Data** | | |
| getLowVoltageArea(...) | Mandatory input arguments:   \| 1 \| userdata \| \| --- \| --- \|   Input parameter-value pairs   \| ‘method’ \| {‘map’} \| ‘egm’ \| \| --- \| --- \| \| ‘type’ \| {‘bip’} \| ‘uni’ \| \| ‘threshold’ \| {[.0 .5]} \| array \|   Output arguments   \| lowVArea \| The low voltage area (cm^2^) \| \| --- \| --- \| \| voltages \| The voltages point values used to calculate areas \| \| iTri \| Indexes into userdata.surface.triRep.Triangulation and refers to the triangles that have voltage values within the range specified by ‘threshold’ \| \| tr2 \| A triangulation of all the triangles referenced in iTri. \| | Use case  Returns the low voltage area  Description  getLowVoltageArea.m Returns the surface area of the chamber with voltage less than the specified threshold, 0.5mV by default. By default, low voltage area is calculated using the surface data (stored in userdat.surface). If 'method' is set to 'egm' then the bipolar voltage is first interpolated from the bipolar electrogram data (stored in userdata.electric). If 'type' is set to 'uni' then unipolar voltages are used for surface area calculation.  Example function call(s)  [lowVArea, voltages, iTri, tr2] = getLowVoltageArea(userdata, 'method', 'egm'); |
| getMeanVoltage(...) | Mandatory input arguments:   \| 1 \| userdata \| \| --- \| --- \|   Input parameter-value pairs   \| ‘method’ \| {‘map’} \| ‘egm’ \| \| --- \| --- \| \| ‘type’ \| {‘bip’} \| ‘uni’ \|   Output arguments   \| meanVoltage \| The mean chamber voltage (in mV) \| \| --- \| --- \| | Use case  Returns the mean voltage of the chamber in userdata  Description  getMeanVoltage.m Returns the mean voltage of a chamber. By default, the mean bipolar voltage is calculated using the interpolated mapping data from the clinical mapping system (stored in userdata.surface.act_bip). If 'method' is set to 'egm' then the bipolar voltage is first interpolated from the bipolar electrogram data (stored in userdata.electric). If 'type' is set to 'uni' then unipolar voltages are returned.  Example function call(s)  meanVoltage = getMeanVoltage( userdata ); |
| getImpedanceValue(...) | Mandatory input arguments:   \| 1 \| userdata \| \| --- \| --- \|   Input parameter-value pairs   \| ‘method’ \| {‘map’} \| ‘egm’ \| \| --- \| --- \| \| ‘points’ \| {‘:’} \| int array \| \| ‘vertices’ \| {‘:’} \| int array \|   Output arguments   \| imp \| The impedance values (Ohms) \| \| --- \| --- \| | Use case  Returns the impedance values of given point(s)  Description  getImpedanceValues.m returns the impedance values. By default, impedance values are returned for all the points in the map. If 'method' is specified to be 'egm' then impedance transients are returned for each individual mapping point, along with time intervals for the impedances. If one or more 'vertices' are specified, then impedance values are only returned for those vertices (only valid if 'method' is 'map'). If one or more 'points' is specified, then impedance values are only returned for those mapping points (only valid if 'method' is 'egm').  Example function call(s)  imp = getImpedanceValues(userdata, 'method', 'egm', 'points', [1 2 3]); |
| voltageHistogramAnalysis(...) | Mandatory input arguments:   \| 1 \| userdata \| \| --- \| --- \|   Input parameter-value pairs   \| ‘method’ \| {‘map’} \| ‘egm’ \| \| --- \| --- \| \| ‘type’ \| {‘bip’} \| ‘uni’ \| \| ‘threshold’ \| {[ 0.01 0.11; 0.11 0.21; 0.21 0.30; 0.30 0.40; 0.40 0.50 ]} \| matrix \| \| ‘plot’ \| {false} \| true \| \| ‘colors’ \| { [colorBrewer('r'); colorBrewer('y'); colorBrewer('g'); colorBrewer('b'); colorBrewer('p')] } \| matrix \|   Output arguments   \| areas \| The chamber areas within each of the voltage thresholds \| \| --- \| --- \| | Use case  Performs voltage histogram analysis  Description  voltageHistogramAnalysis.m displays a histogram of voltages coloured according to voltages, threshold. If 'method' is set to 'egm' then the bipolar voltage is first interpolated from the bipolar electrogram data (stored in userdata.electric). If 'type' is set to 'uni' then unipolar voltages are used.  Example function call(s)  areas = voltageHistogramAnalysis(userdata, 'plot', true, 'method', 'map'); |
| **Visualisation Functions** | | |
| drawMap(...) | Mandatory input arguments:   \| 1 \| userdata \| \| --- \| --- \|   Input parameter-value pairs   \| ‘data’ \| {[]} \| array \| \| --- \| --- \| \| ‘type’ \| {'act'} \| 'bip' \| 'force' \| 'uni' \| 'none' \| 'cv' \| \| ‘coloraxis’ \| {[]} \| array \| \| ‘noLight’ \| {false} \| true \| \| ‘usrColorMap’ \| {[]} \| matrix \| \| ‘colorbarlocation’ \| 'north' \| 'south' \| 'east' \| 'west' \| 'northoutside' \| 'southoutside' \| 'eastoutside' \| {'westoutside'} \| \| ‘orientation’ \| {‘AP’} \| ‘PA’ \| \| ‘colorfillthreshold’ \| {10} \| double \|   Output arguments   \| hSurf \| A handle to the plotted surface \| \| --- \| --- \| | Use case  Plot an OpenEP map  Description  drawMap.m is a wrapper function for colorShell.m which allows an OpenEP map to be plotted.  Example function call(s)  hSurf = drawMap(userdata, 'type', 'act'); |
| plotTag(...) | Mandatory input arguments:   \| 1 \| userdata \| \| --- \| --- \|   Input parameter-value pairs   \| ‘coord’ \| {[]} \| X \| \| --- \| --- \| \| ‘pointnum’ \| {[]} \| p \| \| ‘color’ \| {'r'} \| 'g' \| 'b' \| 'p' \| 'o' \| 'y' \| \| ‘size’ \| 4 \| double \|   Output arguments   \| h \| An array of handles referencing the plotted surfaces \| \| --- \| --- \| | Use case  Plot tag(s) on the current map  Description  Plot tag(s) on the current map  Example function call(s)  h = plotTag( userdata ); |
| pointStatus(...) | Mandatory input arguments:   \| 1 \| userdata \| \| --- \| --- \|   Input parameter-value pairs   \| ‘tol’ \| {0.1} \| double \| \| --- \| --- \| \| ‘plot’ \| {false} \| true \|   Output arguments   \| inoutpts \| Whether points are internal (logical(1)) or external (logical(0)) to the triangulation in userdata \| \| --- \| --- \| \| meshpts \| Whether points in the triangulation in userdata are referenced in the triangulation (logical(1)) or not (logical(0)) \| | Use case  Returns the status of points relevant to userdata  Description  pointStatus depends on the package inpolyhedron. See:  <https://uk.mathworks.com/matlabcentral/fileexchange/37856-inpolyhedron-are-points-inside-a-triangulated-volume>  Example function call(s)  [inoutputs, meshpts] = pointStatus( userdata ); |
| **Ablation Data** | | |
| plotVisitags(...) | Mandatory input arguments:   \| 1 \| userdata \| \| --- \| --- \|   Input parameter-value pairs   \| ‘plot’ \| {‘tags’}\|’grid’\|’both’ \| \| --- \| --- \| \| ‘shell’ \| {‘on’}\|’off’ \| \| ‘colour’ \| {‘r’}\|colorspec\|array \| \| ‘orientation’ \| See drawMap.m \|   Output arguments   \| None \|  \| \| --- \| --- \| | Use case  Displays ablation data for a case  Description  plotVisitags() requires a userdata structure which contains .rfindex as its input, which can be created using importvisitag().  Example function call(s)  plotVisitags(userdata)  plotVisitags(userdata, ‘plot’, ‘both’, ‘shell’, ‘off’, ‘orientation’, ‘ap’); |
| getAblationArea(...) | Mandatory input arguments:   \| 1 \| userdata \| \| --- \| --- \|   Input parameter-value pairs   \| ‘method’ \| {‘tags’}\|’grid’ \| \| --- \| --- \| \| ‘radius’ \| {5}\|double \|   Output arguments   \| ablArea \| The total area of the chamber that has been ablated \| \| --- \| --- \| \| isAblated \| Indexes into userdata.surface.triRep.Triangulation and indicates whether a particular triangle is considered ablated (1) or not (0). \| \| trAbl \| A triangulation of the ablated tissue. \| | Use case  Calculates the area of a chamber which has been ablated  Description  getAblationArea() requires a userdata structure which contains .rfindex as its input, which can be created using importvisitag().  Example function call(s)  [ablArea, isAblated, trAbl] = getAblationArea(userdata); |
| plotAblationArea(...) | Mandatory input arguments:   \| 1 \| userdata \| \| --- \| --- \|   Input parameter-value pairs   \| None \|  \| \| --- \| --- \|   Output arguments   \| None \|  \| \| --- \| --- \| | Use case  Adds the ablation area to the current figure  Description  plotAblationArea() requires a userdata structure which contains .rfindex as its input, which can be created using importvisitag().  Example function call(s)  plotAblationArea(userdata); |
| **Electrogram Data** | | |
| plotOpenEPEgms(...) | Mandatory input arguments:   \| 1 \| userdata \| \| --- \| --- \|   Input parameter-value pairs   \| ‘iegm’ \| {:}\|[a:b] \| \| --- \| --- \| \| ‘range’ \| {‘window’}\|’all’ \| \| ‘buffer’ \| {50}\|double \| \| ‘egmtype’ \| ‘bip’\|’uni’\|{’bip-uni’} \| \| ‘reference’ \| ‘off’\|{‘on’} \|   Output arguments   \| hFig \| A handle to the plotted figure \| \| --- \| --- \| | Use case  Plot electrograms from OpenEP data  Description  plotOpenEPEgms is a wrapper function for plotElectrograms.  Example function call(s)  plotOpenEPEgms(userdata, ‘iegm’, getIndexFromCartoPointNumber(userdata,1)); |
| getEgmsAtPoints(...) | Mandatory input arguments:   \| 1 \| userdata \| \| --- \| --- \|   Input parameter-value pairs   \| ‘iEgm’ \| {:}\|[a:b] \| \| --- \| --- \| \| ‘egmtype’ \| ‘bip’\|’uni’\|{‘bip-uni’} \| \| ‘reference’ \| ‘off’\|{‘on’} \|   Output arguments   \| egmTraces \| Cell array of electrograms \| \| --- \| --- \| \| ‘egmtype’ \| Cell array of activation times \| \| ‘reference’ \| Names of the electrograms \| | Use case  Access electrograms stored in the OpenEP data format  Description  getEgmsAtPoints by default returns all the electrograms of ‘egmtype’. Use getIndexFromCartoPointNumber to convert from point numbers to index numbers.  Example function call(s)  [egmTraces, acttime, egmNames] = getEgmsAtPoint(userdata, ’iEgm’, 1, ‘egmtype’, ’bip’, ’reference’, ‘off’); |
| **Batch Processing Functions** | | |
| batchImport(...) | None | Example script provided to allow batch importing of all datasets in manufacturer-exported format contained within a folder. |
| batchProcess(...) | None | Example script provided to allow batch processing of all datasets in OpenEP format in a folder. |
