# Supplementary material for: OpenEP: A Cross-Platform Electroanatomic Mapping Data Format and Analysis Platform for Electrophysiology Research
Source: Front Physiol. 2021 Feb 26;12:646023. doi: 10.3389/fphys.2021.646023 (PMC7952326; doi:10.3389/fphys.2021.646023)
Supplement: Supplementary file 9 [file Table_3.pdf]

Supplementary Material

Supplementary Table 3: OpenEP Electroanatomic Mapping Data Structure v1.0

Field name	Description	Size
`Userdata`		
`.systemName`	Name of the clinical system that was used for collecting the clinical data	
`.cartoFolder`	Absolute path to the original directory in which a Carto dataset was unzipped.	
`.velocityFolder`	Absolute path to the original directory in which a Velocity dataset was unzipped.	
`.precisionFolder`	Absolute path to the original directory in which a Precision dataset was unzipped.	
`.rhythmiaFolder`	Absolute path to the original directory in which a Rhythmia dataset was unzipped.	
`.electric`		
`.tags`	The physician-visible names of the points applied during the clinical case.	n points x 1
`.names`	The internal names of the points used by the clinical electroanatomic mapping system	n points x 1
`.electrodeNames_bip`	The names of the electrode pair recording the bipole electrogram at each point	n points x 1
`.egmX`	The Cartesian co-ordinates of the mapping point	n points x 3
`.egm`	The recorded bipolar elecrtograms	n points x egm duration
`.eletrodeNames_uni`	The names of the two unipole electrodes which make up the bipolar electrode pair at each mapping point.	n points x 2
`.egmUniX`	The Cartesian co-ordinates of the two unipole electrodes which make up the bipolar electrode pair at each mapping point.	n points x 3 x 2
`.egmUni`	The recorded unipolar electrograms	n points x egm duration x 2
`.egmRef`	The reference electrograms for each point	n points x egm duration
`.ecg`	The surface electrocardiogram for each point	n points x egm duration
`.sampleFrequency`	The sampling frequency of electrogrms in the dataset	Integer (Hz)
`.annotations`		
`.woi`	The window of interest for each point relative to the reference annotation	n points x 2
`.referenceAnnot`	The reference annotation for each point with one value each for the start and end of the window	n points x 1

.mapAnnot	The local activation time annotation for each point relative to the reference annotation	n points x 1
.signalProps{1..m}	Sub-field to contain data relating to each of *m* clinical system-defined signal properties	m signal properties
.name	Name of the clinical-system derived signal property metric (for example CFAEMean)	n points x 1
.value	Value of the signal property for each of n points	n points x 1
.propSettings	String containing the user-defined settings used to create the clinical system-derived signal properties, for example "peak amplitude,*X;* temporal_spacing,*Y*"	Variable length string
.voltages		
.bipolar	The bipolar voltage measured by the clinical mapping system for each point	n points x 1
.unipolar	The unipolar voltage measured by the clinical mapping system for each point	n points x 1
.impedances		
.time	Cell array of time values for impedance traces for each point	1 x n points
.time{n}	Time values for impedance traces for point n	Impedance trace duration x 1
.value	Cell array of impedance values for for each point	1 x n points
.value{n}	Impedance values for point n	Impedance trace duration x 1
.egmSurfX	The Cartesian co-ordinates of the mapping point, projected to the closest point on the surface of the chamber	n points x 3
.barDirection	Surface normal for each mapping point	n points x 3
.notes	Cell array of notes relevant to the dataset	>=1 x n notes
.surface		
.triRep	A Trirep object	
.X	The Cartesian co-ordinates of the vertices in the surface mesh	n vertices x 3
.Triangulation	The vertex indices of each face in the surface mesh	n faces x 3
.isVertexAtRim	Logical array with true values indicating vertices that are on a free boundary	n vertices x 1
.act_bip	Local activation time data (column 1) and bipolar voltage data (column 2) from the clinical map	n vertices x 2
.uni_imp_frc	Unipolar voltage data (column 1), impedance data (column 2) and force data (column 3) from the clinical map	n vertices x 3
.signalMaps{1..n}	Sub-field to contain data relating to each of *m* clinical system-defined signal property maps	m signal property maps

.name	Name of the clinical-system derived signal property map (for example CFAEMean)	Single string value
.map	Value of the signal properties at every point	n points x 1
.propSettings	String containing the user-defined settings used to create the clinical system-derived signal properties, for example “`peak amplitude,X; temporal_spacing,Y`”	Variable length string
.rf	Structure field to store manually acquired ablation site data	
.originaldata		
.force	Force data	
.time	Ablation time data	n time intervals x 1
.force	Contact force data	n time intervals x 1
.axialangle	Axial angle	n time intervals x 1
.lateralangle	Lateral angle	n time intervals x 2
.position	Cartesian co-ordinates	n time intervals x 3
.ablparams	Ablation data	
.time	Ablation time data	n time intervals x 1
.power	Power	n time intervals x 1
.impedance	Impedance	n time intervals x 1
.distaltemp	Temperature	n time intervals x 1
.rfindex	Structure field to store automatically acquired ablation site data	
.name		
.tag	Structure field to store ablation tag data from the Carto3 Visitag module	
.X	Cartesian co-ordinates of each tag	n tag x 3
.time	Time stamp of each tag	n tag x 1
.avgForce	Average force applied at each tag	n tag x 1
.maxTemp	Maximum recorded catheter tip temperature at each tag	n tag x 1
.maxPower	Maximum applied radiofrequency power at each tag	n tag x 1
.Impedance		
.baseImp	Baseline impedance, in Ohms, recorded at each tag	n tag x 1
.impDrop	Impedance drop, in Ohms, recorded at each tag	n tag x 1
.fti	Force time integral calculated at each tag	n tag x 1
.index		
.name	Proprietary name of the calculated radiofrequency index	String
.value	Value of the proprietary radiofrequency index	n tag x 1
.grid	Structure field to store grid data from the Carto3 Visitag module	n tag x 1 cell array

		containing m grid point x 1 array
`.X`	Cartesian co-ordinates of this grid point in the ablation dataset [X, Y, Z]	1 x 3 array
`.time`	Timeseries for this grid point	n time intervals x 1
`.index`		
`.name`	Proprietary name of the calculated radiofrequency index	String
`.value`	Timeseries of the proprietary radiofrequency index	n time intervals x 1
`.impedance`	Impedance timeseries for this grid point, Ohms	n time intervals x 1
`.temperature`	Temperature timeseries for this grid point, C	n time intervals x 1
`.power`	Power timeseries for this grid point, W	n time intervals x 1
`.impedanceDrop`	Impedance drop timeseries for this grid point, Ohms	n time intervals x 1
`.force`	Force timeseries for this grid point, g	n time intervals x 1
